# Supplementary material for: The Sensory Coding of Warm Perception
Source: Neuron. 2020 Jun 3;106(5):830–841.e3. doi: 10.1016/j.neuron.2020.02.035 (PMC7272120; doi:10.1016/j.neuron.2020.02.035)
Supplement: Document S1. Figures S1–S7 [file mmc1.pdf]

Neuron, Volume *106*

Supplemental Information

The Sensory Coding of Warm Perception

Ricardo Paricio-Montesinos, Frederick Schwaller, Annapoorani Udhayachandran, Florian Rau, Jan Walcher, Roberta Evangelista, Joris Vriens, Thomas Voets, James F.A. Poulet, and Gary R. Lewin

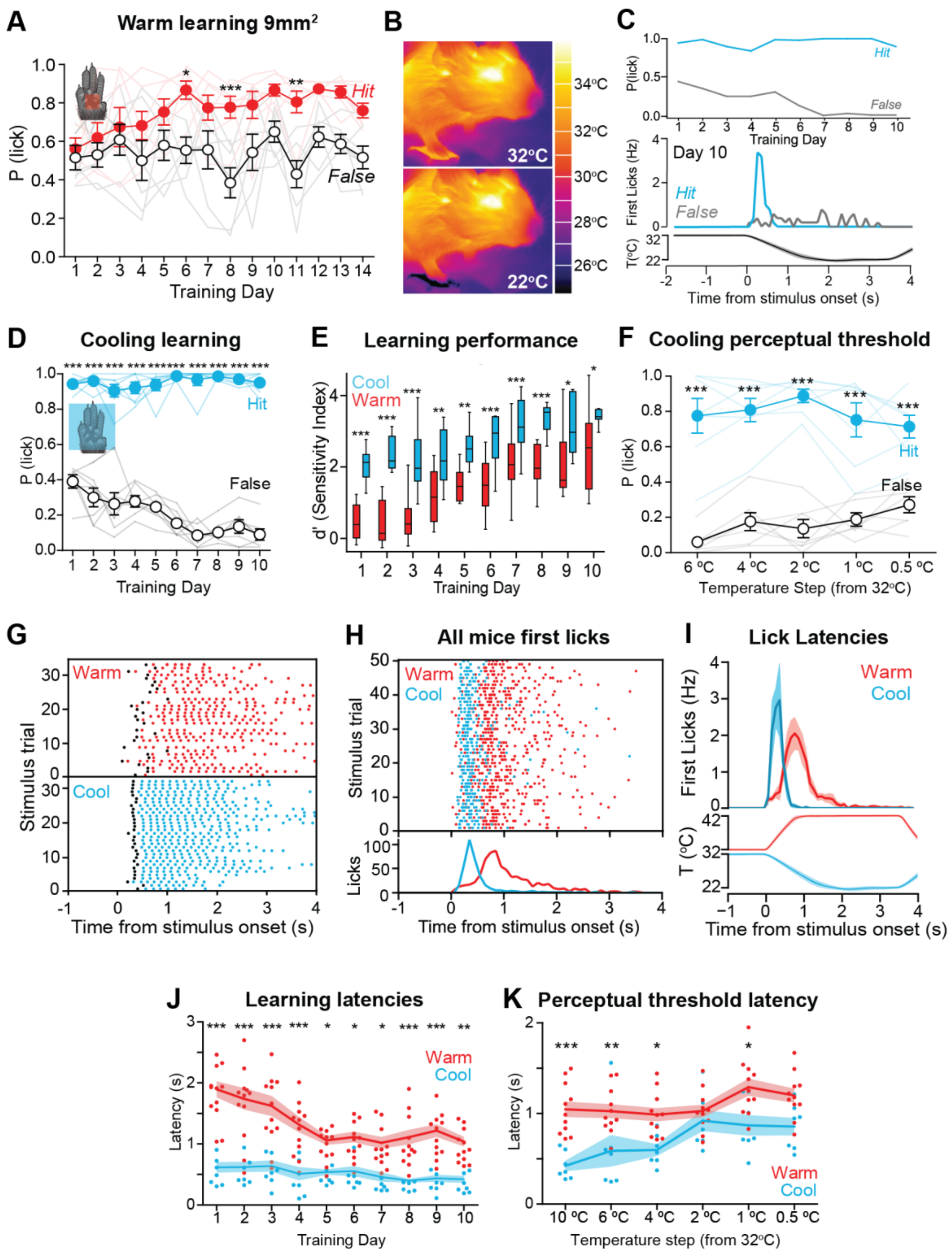

Figure S1 – Related to Figure 1 Comparison Warm vs Cool

(A) Wild type mice did not learn to reliably report warm stimuli of 32-42˚C (delivered by a 3x3mm Peltier) after 14 training sessions (n = 7; two-way repeated measures ANOVA with Bonferroni post-hoc tests). (B) Wild type mice were trained to report cool stimuli of 32-22 ˚C delivered to the right forepaw. (C) Representative learning curve (top) and lick latency distribution at training day 10 (bottom) of a cool-trained mouse using an 8x8 mm Peltier element. (D) Wild type mice learnt to report cool stimuli of 32-22˚C(delivered by a 8x8mm Peltier) from the 1st training session (n = 7; two-way repeated measures ANOVA with Bonferroni post-hoc tests). (E) Sensitivity (d') was higher for cool- than for warm-trained mice across training sessions (n = 7 cool, n = 12 warm; two-way repeated measures ANOVA with Bonferroni post-hoc tests). (F) Decreasing stimulus amplitude over

consecutive training sessions revealed a perceptual threshold of 0.5°C (n = 6; two-way repeated measures ANOVA with Bonferroni post-hoc tests). (G) Lick latencies of a representative warm-trained (top) and cool-trained (bottom) mice on the last training session. The first lick to respond to each stimulus (latency) is shown in black, and the rest of the licks are shown in red (warm) or blue (cool). (H) Plot of all lick latencies from all mice on the last training session for cool and warm shows a higher spread and longer latency of the licks in warm-trained mice. (I) First lick latency PSTH of all warm- and cool-trained mice on their fastest session (chosen from sessions of good performance, with d' > 1.5) (top). Average temperature trace during warm and cool detection sessions show very similar Peltier dynamics during cooling and warming (bottom). (J) Session average lick latencies were slower for warm than for cool stimuli across training sessions (n = 12 warm, n = 7, two-way repeated measures ANOVA with Bonferroni post-hoc tests).

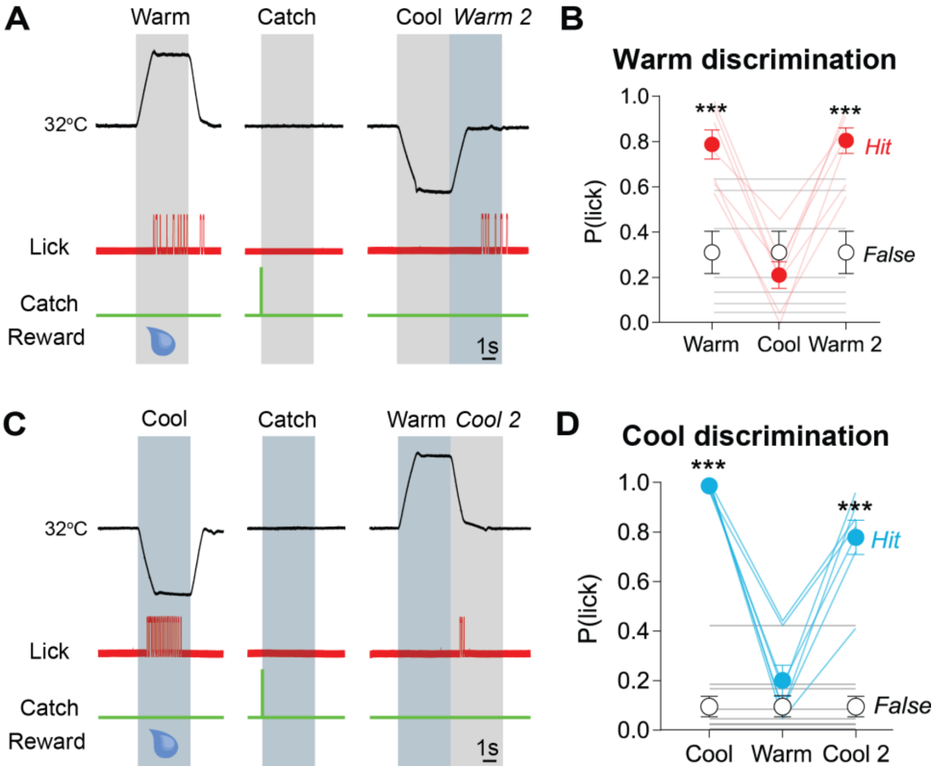

Figure S2 – Related to Figure 1 Discrimination task

(A) Scheme of the thermal discrimination task for warm-trained mice. Cool trials were introduced, and no reward was given if mice licked during cool stimuli. Licks were also assessed during the warming phase of the right after the cool stimulus ("Warm 2"). (B) Warm-trained mice licked the sensor during both warm types, but not during cool stimuli (n = 7, hit vs false; two-way repeated measures ANOVA with Bonferroni post-hoc tests).

(C) Scheme of the thermal discrimination task for cool-trained mice. Warm trials were introduced, and no reward was given if mice licked during warm stimuli. Licks were also assessed during the cool phase right after the cool stimulus ("Cool 2"). (D) Cooling-trained mice could correctly discriminate cooling from warm, and reported cool regardless of the absolute temperature (n = 7; hit vs false two-way repeated measures ANOVA with Bonferroni post-hoc tests). *P < 0.05, **P < 0.01, ***P < 0.001. Data = mean ± SEM.

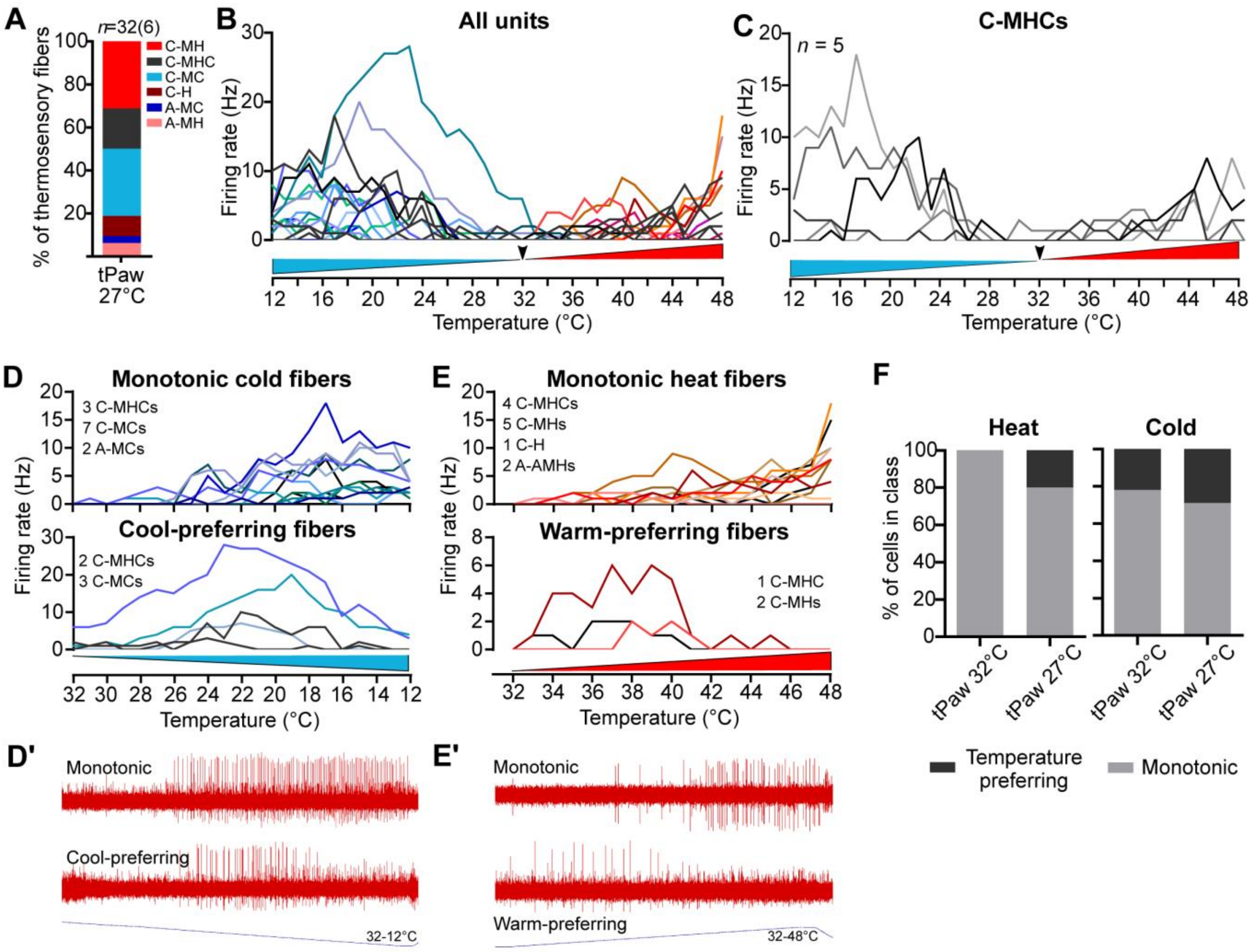

Figure S3 – Related to Figure 2 Additional skin-nerve data at bath 27˚C

(A) Proportions of thermosensory C-fibers in control mice recorded from the forepaw skin with bath temperature at 27 ˚C. Total animals recorded from shown in brackets. (B) Firing activity of all individual fibers during 32-48ºC and 32-12ºC ramps. Arrow marks stimulus starting point. (C) Firing activity of all individual C-mechanoheatcold (C-MHC) fibers during 32-48˚C and 32-12˚C ramps. (D) Cold-sensitive fibers were either monotonic (top) and increased firing activity linearly during 32-12˚C ramp, or were cool-preferring (bottom) and preferentially fired during the cool phase of the ramp. (D') Example traces recorded from a monotonic (top) and a cool-preferring (bottom) fiber. (E) Heat-sensitive fibers were either monotonic (top) and increased firing activity linearly during 32-48 ˚C ramp, or were warm-preferring (bottom) and preferentially fired during the warm phase of the ramp. (E') Example traces recorded from a monotonic (top) and a warm-preferring (bottom) fiber. (F) Proportions of heat and cold fiber subtypes recorded when the temperature of the paw was set to 32 ˚C or 27˚C.

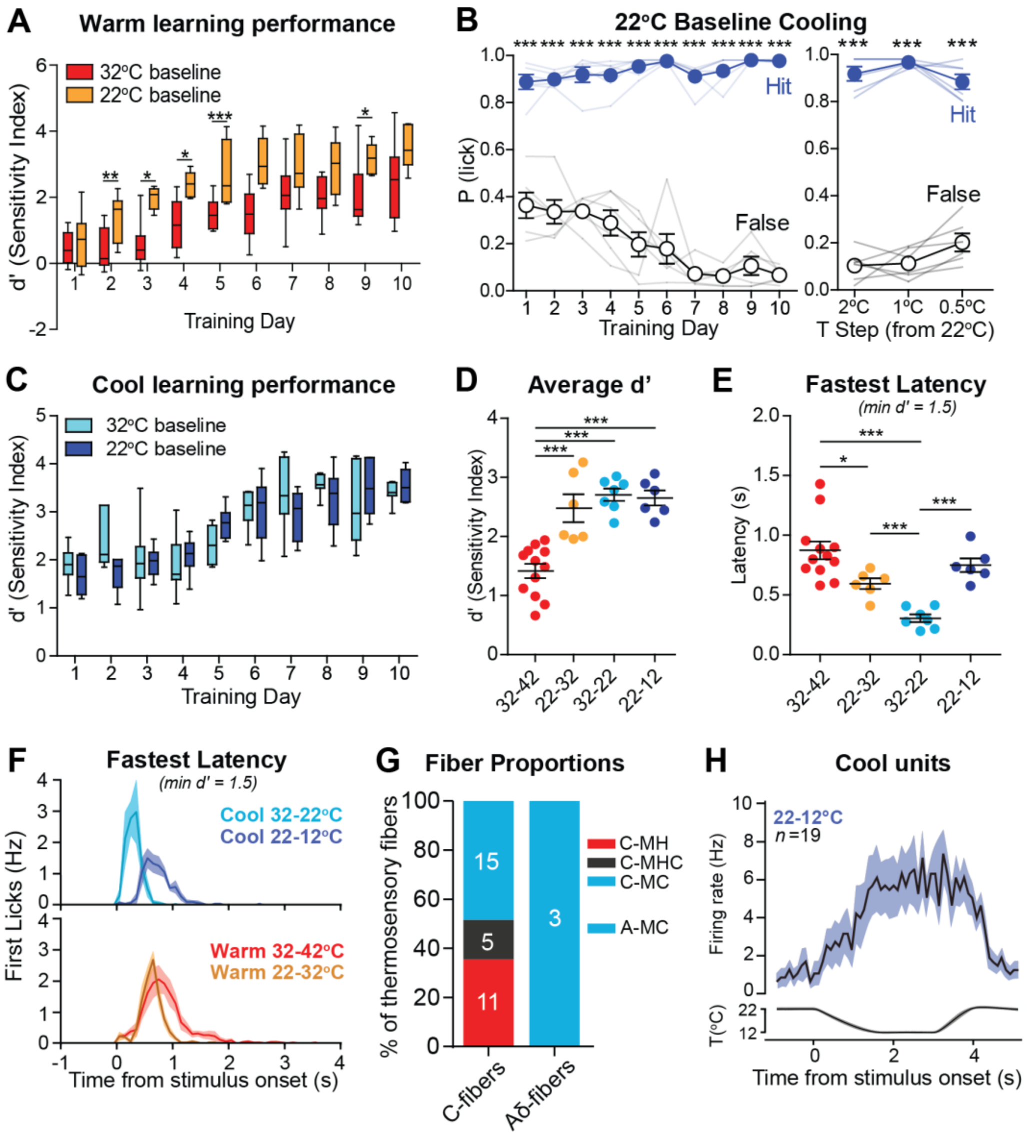

Figure S4 – Related to Figure 3 Additional data 22˚C baseline

(A) Sensitivity (d') of WT mice trained to report warm of 32-42˚C (n = 12) and 22-32˚C (n = 6). Mice trained to report warm of 22-32˚C had an overall slightly better performance than mice trained at 32-42˚C (two-way repeated measures ANOVA with Bonferroni post-hoc). (B) WT mice could report cool of 22-12ºC (n = 6), as well as cool stimuli of 0.5˚C starting from 22˚Cbaseline (n = 6) (hit vs false two-way repeated measures ANOVA with Bonferroni post-hoc). (C) Sensitivity (d') of WT mice trained to report cool of 32-22˚C (n = 7) was overall very similar to that of mice trained to report cool of 22-12˚C (n = 6) (two-way repeated measures ANOVA with Bonferroni post-hoc). (D) Average sensitivity (d') across all training sessions was lower for mice trained to report warming at 32-42˚C (n = 12, d' = 1.42 ± 0.12) than for warming at 22-32˚C (n = 6, d' = 2.48 ± 0.24, p = 0.0004 vs 32-42˚C), cool at 32-22˚C (n = 7, d' = 2.71 ± 0.11, p < 0.0001 vs 32-42˚C) and cooling at 22-12˚C (n = 6, d' = 2.65 ± 0.13, p < 0.0001 vs 32-42˚C). (E) The fastest latency achieved across all training sessions (with good performance, d' at least 1.5) was slower for WT mice trained to report warm at 32-42˚C (n = 12, mean 0.87 ± 0.07s) than for warm at 22-32˚C (n = 6, mean 0.59 ± 0.04s, p = 0.023 vs 32-42˚C). However, cool steps of 32-22˚C (n = 7, mean 0.31 ± 0.03s) could be reported quicker than cool steps of 22-12˚C (n = 6, mean 0.75 ± 0.06s, p < 0.0001 vs 32-22˚C). Also, cool step at 32-22˚C could be reported faster than warm at 32-42˚C (p < 0.0001). (F) Mean hit and false alarm lick PSTHs of WT mice trained to report cool steps of either 32-22 (n = 7) or 22-12˚C (n = 6) (top), and warm steps of 32-42 (n = 12) or 22-32˚C (n = 6) (bottom). (G) Fiber proportions found in skin-nerve sensory afferent recordings of forepaw in the 22˚C Peltier baseline experiments (and tissue kept in a bath at 27˚C) (n = 34 fibers). Fibers were screened using a slow ramp between 12 and 42˚C. (H) PSTH of

sensory afferent action potential responses to 4-second cool steps at 22-12°C. *P < 0.05, **P < 0.01, ***P < 0.001. Data = mean ± SEM.

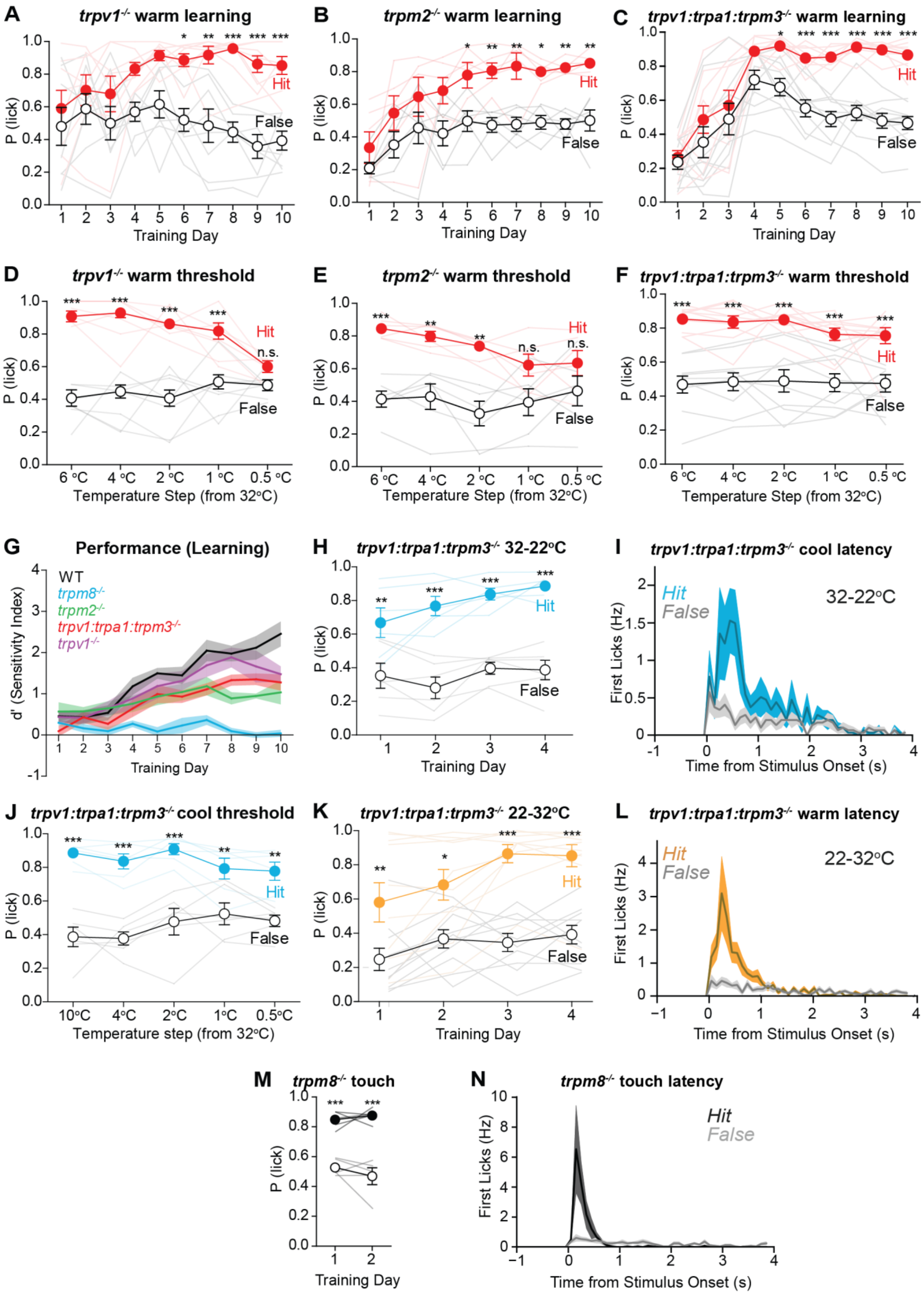

A
trpv1-/- warm learning
P (lick)
Training Day
Hit
False
B
trpm2-/- warm learning
P (lick)
Training Day
Hit
False
C
trpv1:trpa1:trpm3-/- warm learning
P (lick)
Training Day
Hit
False
D
trpv1-/- warm threshold
P (lick)
Temperature Step (from 32°C)
6 °C 4 °C 2 °C 1 °C 0.5 °C
Hit
False
n.s.
E
trpm2-/- warm threshold
P (lick)
Temperature Step (from 32°C)
6 °C 4 °C 2 °C 1 °C 0.5 °C
Hit
False
n.s.
F
trpv1:trpa1:trpm3-/- warm threshold
P (lick)
Temperature Step (from 32°C)
6 °C 4 °C 2 °C 1 °C 0.5 °C
Hit
False
G
Performance (Learning)
d' (Sensitivity Index)
Training Day
WT
trpm8-/-
trpm2-/-
trpv1:trpa1:trpm3-/-
trpv1-/-
H
trpv1:trpa1:trpm3-/- 32-22°C
P (lick)
Training Day
Hit
False
I
trpv1:trpa1:trpm3-/- cool latency
First Licks (Hz)
Time from Stimulus Onset (s)
Hit
False
32-22°C
J
trpv1:trpa1:trpm3-/- cool threshold
P (lick)
Temperature step (from 32°C)
10°C 4°C 2°C 1°C 0.5°C
Hit
False
K
trpv1:trpa1:trpm3-/- 22-32°C
P (lick)
Training Day
Hit
False
L
trpv1:trpa1:trpm3-/- warm latency
First Licks (Hz)
Time from Stimulus Onset (s)
Hit
False
22-32°C
M
trpm8-/- touch
P (lick)
Training Day
N
trpm8-/- touch latency
First Licks (Hz)
Time from Stimulus Onset (s)
Hit
False

Figure S5 – Related to Figure 5 Additional *trp* data and WT Comparison

(A) *trpv1*$^{-/-}$ mice learnt to report warm stimuli of 32-42°C delivered to the forepaw (n = 8, hit vs false two-way repeated measures ANOVA with Bonferroni post-hoc tests). (B) *trpm2*$^{-/-}$ mice learnt to report warm stimuli of 32-42°C delivered to the forepaw (n = 6, hit vs false two-way repeated measures ANOVA with Bonferroni post-hoc tests). (C) *trpv1:trpa1:trpm3*$^{-/-}$ mice learnt to report warm stimuli of 32-42°C delivered to the forepaw (n = 10, hit vs false two-way repeated measures ANOVA with Bonferroni post-hoc tests). (D) *trpv1*$^{-/-}$ mice could report warm stimuli of as little as 1°C (n = 8, hit vs false two-way repeated measures ANOVA with Bonferroni post-hoc tests). (E) *trpm2*$^{-/-}$ mice could report warm stimuli of as little as 2°C (n = 6, hit vs false two-way repeated measures ANOVA with Bonferroni post-hoc tests). (F) *trpv1:trpa1:trpm3*$^{-/-}$ mice could also report very small warm stimuli as indicated by statistical differences between hit and false alarms, although false alarms were particularly high in this group (n = 10, hit vs false two-way repeated measures ANOVA with Bonferroni post-hoc tests). (G) Sensitivity (d') measurements of warm detection over training sessions for WT, *trpv1*$^{-/-}$, *trpm2*$^{-/-}$, *trpv1:trpa1:trpm3*$^{-/-}$ and *trpm8*$^{-/-}$ mice show that WT mice, as well as the heat-activated *trp* mutant lines had performances above chance level (d' = 0) but *trpm8*$^{-/-}$ mice remained close to d' = 0 across all sessions. (H) *trpv1:trpa1:trpm3*$^{-/-}$ mice learnt to report cool stimuli of 32-22°C delivered to the forepaw (n=6, hit vs false two-way repeated measures ANOVA with Bonferroni post-hoc tests). (I) First lick PSTH of *trpv1:trpa1:trpm3*$^{-/-}$ mice during session 4 of cool detection (32-22°C). (J) *trpv1:trpa1:trpm3*$^{-/-}$ mice could report tiny cool stimuli delivered to the forepaw (n = 6, hit vs false two-way repeated measures ANOVA with Bonferroni post-hoc tests). (K) *trpv1:trpa1:trpm3*$^{-/-}$ mice could report cool stimuli of 22-32°C delivered to the forepaw (n = 10, hit vs false two-way repeated measures ANOVA with Bonferroni post-hoc tests). (L) First lick PSTH of *trpv1:trpa1:trpm3*$^{-/-}$ mice during session 4 of 22-32°C warm detection. (M) Despite being unable to report warm stimuli, *trpm8*$^{-/-}$ mice could report tactile stimuli delivered to their forepaw (n = 5, hit vs false two-way repeated measures ANOVA with Bonferroni post-hoc tests). (N) First lick PSTH of *trpm8*$^{-/-}$ mice during session 2 of tactile detection shows that mice reported the tactile stimulus with high precision (n = 5). *P < 0.05, **P < 0.01, ***P < 0.001. Data = mean ± SEM.

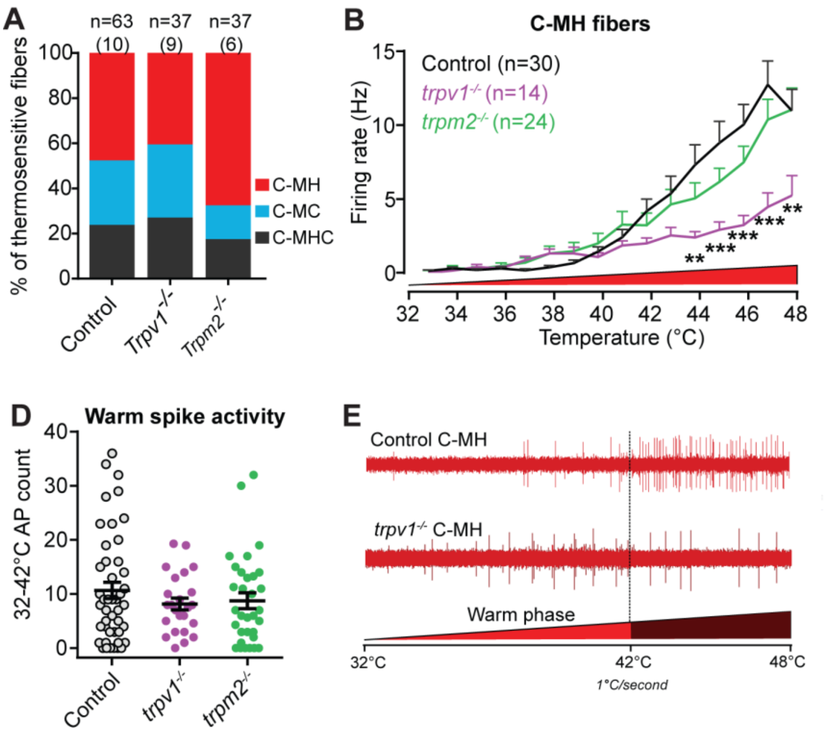

Figure S6 – Related to Figure 5 *trpv1*$^{-/-}$ and t*rpm2*$^{-/-}$ skin-nerve recordings

(A) Proportions of thermosensory C-fibers in control, *trpv1*$^{-/-}$ and t*rpm2*$^{-/-}$ mice recorded from the hindpaw skin with bath temperature at 32ºC. Total number of animals recorded from in each group shown in brackets. (B) Firing activity of C-mechanoheat (C-MH) fibers during 32-48°C heat ramp. C-MH fibers recorded in *trpv1*$^{-/-}$ mice had significantly lower spike activity compared to control fibers from 43-48 °C (Repeated measures Two-way ANOVA with Bonferroni post-hoc test). (C) Firing activity of C-mechanoheatcold (C-MHC) fibers during 32-48 °C heat ramp. (D) Total spike activity of heat-evoked fibers during the warming phase of the ramp (32-42°C). (E) Example recording traces from a control C-MH and a *trpv1*$^{-/-}$ C-MH fiber during heat ramp. (F) Firing activity of all cold-sensitive fibers during 32-12°C cold ramp. $^{**}P < 0.01$, $^{***}P < 0.001$. Data = mean ± SEM.

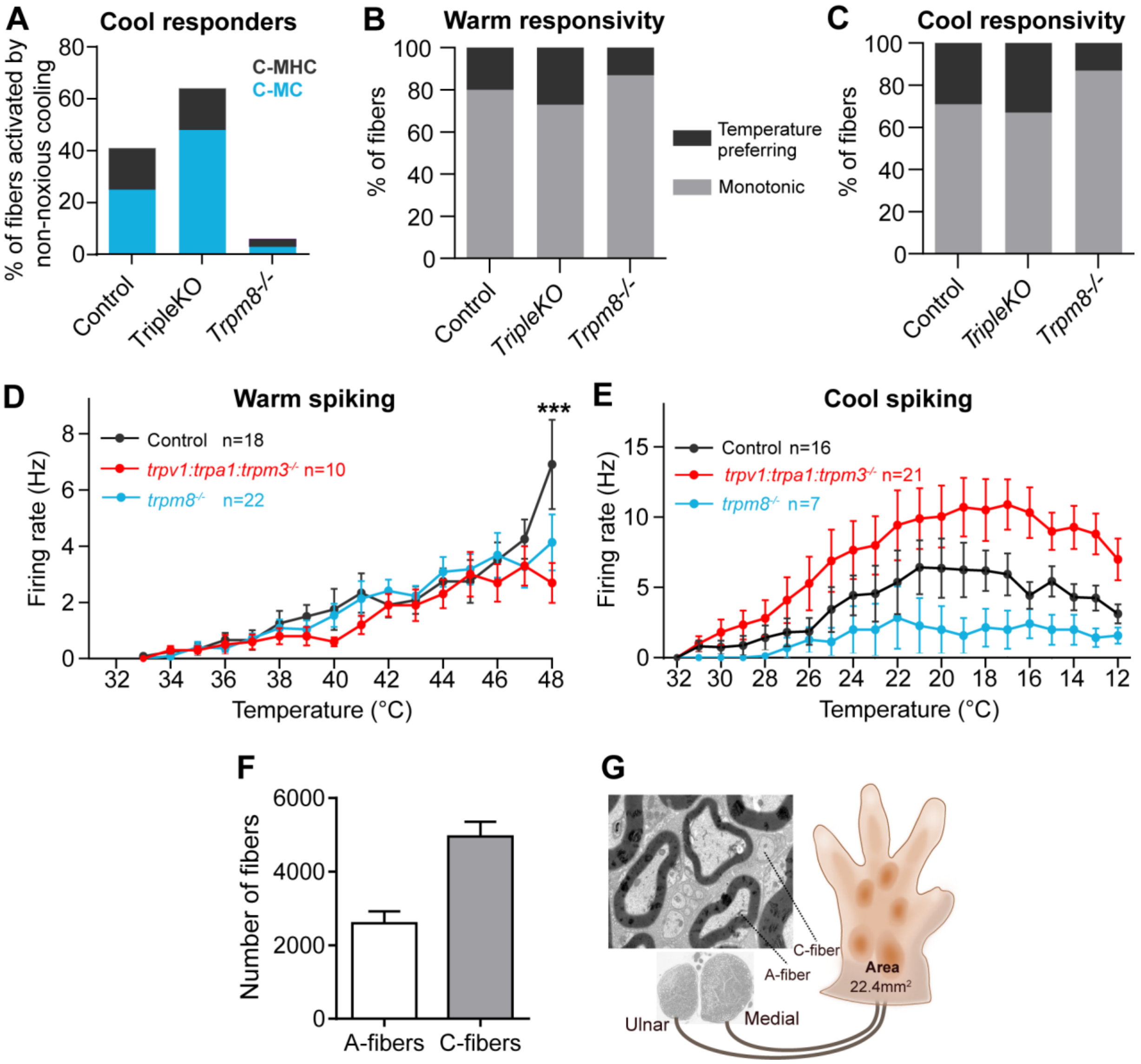

Figure S7 – Related to Figure 7 Additional t*rpm8*$^{-/-}$ and *trpv1:trpa1:trpm3*$^{-/-}$ recording data

(A) Proportions of fibers that were responsive to non-noxious cooling. (B) Proportions of heat-monotonic or warm-preferring units. (C) Proportions of cold-monotonic or cool-preferring units. (D) Firing activity of heat-responsive fibers during 32-48˚C heat ramp. Fibers recorded in *trpv1:trpa1:trpm3*$^{-/-}$ mice had significantly lower spike activity compared to control fibers at 48˚C (Repeated measures Two-way ANOVA with Bonferroni post-hoc test). (E) Firing activity of cold-responsive fibers during 32-12˚C heat ramp. (F) Number of A-fibers and C-fibers that innervate the forepaw, estimated via electron microscopy (n = 4). (G) Example electron micrograph of afferent myelinated (A-type) and unmyelinated (C-type) fibers of the medial and ulnar nerves, which innervate the forepaw skin area. ***P < 0.001. Data = mean ± SEM.
