# Supplementary material for: The Sensory Coding of Warm Perception
Source: Neuron. 2020 Jun 3;106(5):830–841.e3. doi: 10.1016/j.neuron.2020.02.035 (PMC7272120; doi:10.1016/j.neuron.2020.02.035)
Supplement: Document S2. Article plus Supplemental Information [file mmc3.pdf]

Neuron

Article

The Sensory Coding of Warm Perception

Graphical Abstract

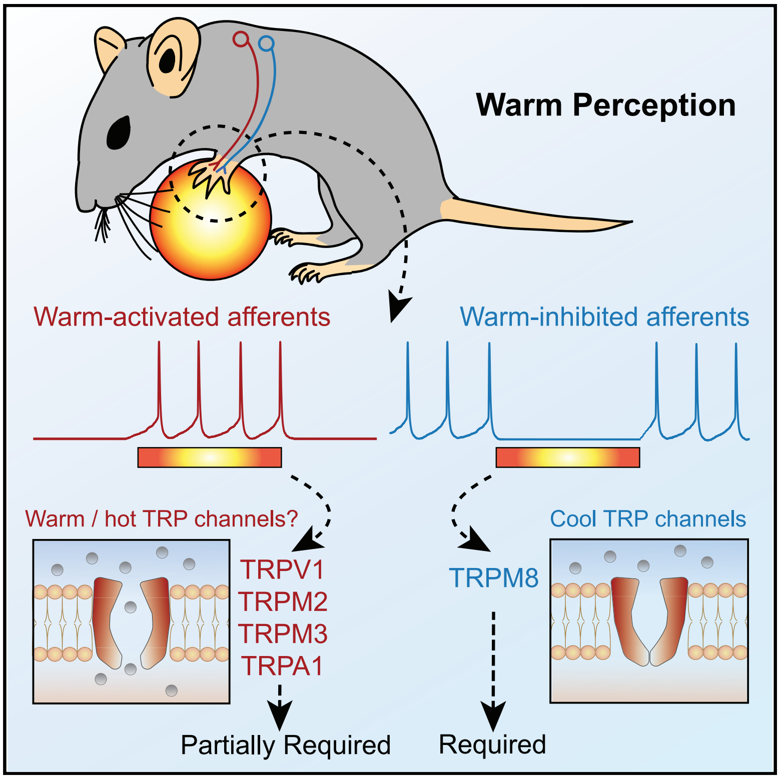

Authors

Ricardo Paricio-Montesinos,
Frederick Schwaller,
Annapoorani Udhayachandran, ...,
Thomas Voets, James F.A. Poulet,
Gary R. Lewin

Correspondence

james.poulet@mdc-berlin.de (J.F.A.P.),
glewin@mdc-berlin.de (G.R.L.)

In Brief

Humans and mice perceive tiny increases in skin temperature as warming. Paricio-Montesinos et al. show that information combined from two types of sensory receptors enables discrimination of warm from cool. Paradoxically, a cool-activated menthol receptor is required for the detection and perception of skin warming.

Highlights

- Mice, like humans, perceive forepaw warming ($\geq 1^\circ$C) and discriminate warm from cool
- Warm-activated and warm-silenced polymodal C-fibers both signal forepaw warming
- Mice lacking the cool-sensitive ion channel TRPM8 are unable to perceive warm
- The inability to perceive warm is associated with loss of warm-silenced C-fibers

Paricio-Montesinos et al., 2020, Neuron *106*, 830–841
June 3, 2020 © 2020 The Author(s). Published by Elsevier Inc.
https://doi.org/10.1016/j.neuron.2020.02.035

CellPress

Neuron

Article

The Sensory Coding of Warm Perception

Ricardo Paricio-Montesinos,[1,2,5] Frederick Schwaller,[1,5] Annapoorani Udhayachandran,[1,2] Florian Rau,[1,2] Jan Walcher,[1] Roberta Evangelista,[1] Joris Vriens,[3] Thomas Voets,[4] James F.A. Poulet,[1,2,*] and Gary R. Lewin[1,6,*]
[1]Department of Neuroscience, Max Delbrück Center for Molecular Medicine, Robert-Rössle Straße 10, 13092 Berlin, Germany
[2]Neuroscience Research Center and Cluster of Excellence NeuroCure, Charité-Universitätsmedizin, Charitéplatz 1, 10117 Berlin, Germany
[3]Laboratory of Endometrium, Endometriosis and Reproductive Medicine, KU Leuven Department of Development and Regeneration, G-PURE, Leuven, Belgium
[4]Laboratory of Ion Channel Research, VIB-KU Leuven Center for Brain and Disease Research, KU Leuven Department of Cellular and Molecular Medicine, Leuven, Belgium
[5]These authors contributed equally
[6]Lead Contact
*Correspondence: james.poulet@mdc-berlin.de (J.F.A.P.), glewin@mdc-berlin.de (G.R.L.)
https://doi.org/10.1016/j.neuron.2020.02.035

SUMMARY

Humans detect skin temperature changes that are perceived as warm or cool. Like humans, mice report fore-paw skin warming with perceptual thresholds of less than 1°C and do not confuse warm with cool. We identify two populations of polymodal C-fibers that signal warm. Warm excites one population, whereas it suppresses the ongoing cool-driven firing of the other. In the absence of the thermosensitive TRPM2 or TRPV1 ion channels, warm perception was blunted, but not abolished. In addition, *trpv1:trpa1:trpm3*$^{-/-}$ triple-mutant mice that cannot sense noxious heat detected skin warming, albeit with reduced sensitivity. In contrast, loss or local pharmacological silencing of the cool-driven TRPM8 channel abolished the ability to detect warm. Our data are not reconcilable with a labeled line model for warm perception, with receptors firing only in response to warm stimuli, but instead support a conserved dual sensory model to unambiguously detect skin warming in vertebrates.

INTRODUCTION

Since the discovery of hot and cold spots on the skin (Blix, 1882), the perception of innocuous warm or cool has been hypothesized to be mediated by specific and separate sensory channels (Schepers and Ringkamp, 2010). Dedicated primary afferent thermoreceptors have been described in primate and human skin that respond exclusively to temperature and fire specifically to cooling or warming, but not painful, thermal stimuli (Campero et al., 2001; Hallin et al., 1982; LaMotte and Campbell, 1978). These afferents typically show ongoing activity at room temperature that is suppressed or enhanced by small temperature changes. Dedicated thermoreceptors have unmyelinated C-fiber axons (Darian-Smith et al., 1979a, 1979b; Susser et al., 1999; Yarnitsky and Ochoa, 1991), but cooling-responsive afferents with thinly myelinated Aδ-axons have also been described (Campero and Bostock, 2010; Darian-Smith et al., 1973; Iggo, 1969; Susser et al., 1999). Warm or cool sensation could also be relayed by polymodal C-fiber afferents that are also mechanosensitive. In contrast to dedicated thermoreceptors, these fibers increase their firing rates monotonically as temperatures become noxious (Campero et al., 1996). The relative contribution of dedicated thermoreceptors as opposed to polymodal temperature-sensitive afferents to the perception of innocuous cool or warm has yet to be addressed.

Recently, it was shown that mice perceive low-threshold thermal stimuli as assessed with a goal-directed perception task (Milenkovic et al., 2014; Yarmolinsky et al., 2016). Mice are able to detect cooling of the skin with perceptual thresholds of just 1°C, similarly to humans (Frenzel et al., 2012; Milenkovic et al., 2014; Stevens and Choo, 1998). We found that activity in polymodal C-fibers was required to perceive innocuous skin cooling (Milenkovic et al., 2014). It is clear that thermosensitive TRP channels are key players in conferring temperature sensitivity to polymodal nociceptors (Caterina et al., 1997; Vandewauw et al., 2018). The availability of mice in which specific *trp* genes have been deleted allows the experimental manipulation of afferent temperature sensitivity to probe the nature of the sensory information required for temperature perception.

At the molecular level, there is overwhelming evidence that the cold-activated ion channel TRPM8 is necessary for the transduction of cold (McKemy, 2013; McKemy et al., 2002); mice lacking this channel have severe noxious and innocuous cool-evoked behavioral and perceptual deficits (Bautista et al., 2007; Dhaka et al., 2007; Knowlton et al., 2013; Milenkovic et al., 2014). Much less is known about candidate molecules for warm transduction; early studies implicated TRPV3 and TRPV4 (Lee et al., 2005; Moqrich et al., 2005), but later studies with mutant mice on pure genetic backgrounds did not support the initial conclusions (Huang et al., 2011). More recently, the TRPM2 channel was shown to be activated by warm temperatures (>35°C) and was implicated as a warm transducer in sensory neurons (Tan and McNaughton, 2016; Togashi et al., 2006; M. Mulier, I. Vandewauw, J.V., T.V., unpublished data). Additionally, the capsaicin and noxious heat-activated TRPV1 channel, which is co-expressed with TRPM2 in sensory neurons (Tan and McNaughton, 2016), was implicated

 © 2020 The Author(s). Published by Elsevier Inc.
This is an open access article under the CC BY license (http://creativecommons.org/licenses/by/4.0/).

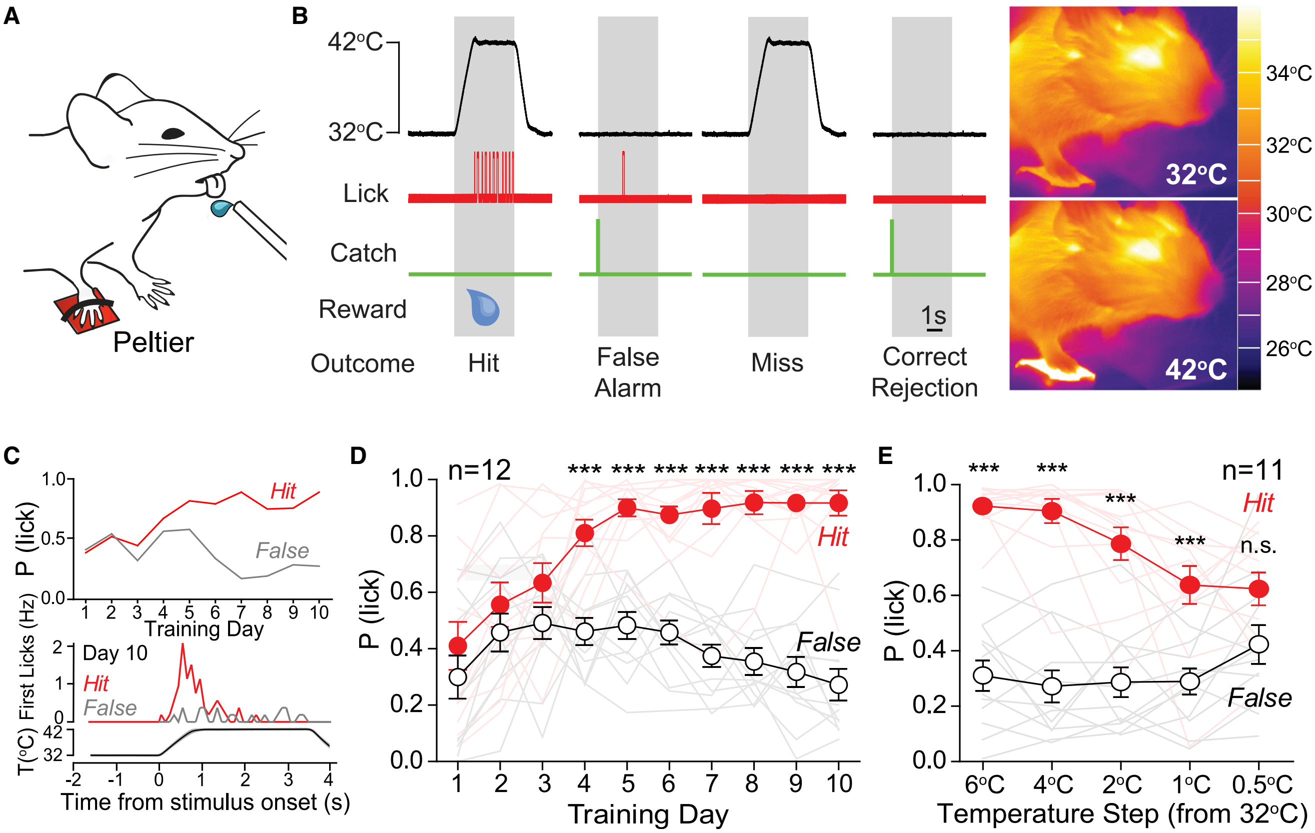

Figure 1. Mice Learn to Report Non-noxious Warm Stimuli Delivered to the Forepaw

(A) Cartoon showing behavioral setup with right forepaw tethered to an 8 × 8 mm Peltier.

(B) Warm-detection task. Temperature baseline was 32°C and reached 42°C for 4 s. Licks within the warming or warm plateau phase (gray area) were water rewarded (hit). Catch trials were introduced with no warm stimulus and used to measure spontaneous licking (false alarms). Right: thermal images of mice with their forepaw resting on the Peltier element.

(C) Example learning curve (top) and PSTH of lick timing at training day 10 (bottom) from one warm-trained mouse.

(D) Mice learned to report warm stimuli of 32°C–42°C after the fourth training session (n = 12; two-way repeated-measures ANOVA with Bonferroni post hoc tests).

(E) Decreasing stimulus amplitude revealed a perceptual threshold of 1°C (n = 11; two-way repeated-measures ANOVA with Bonferroni post hoc tests).

*p < 0.05, **p < 0.01, and ***p < 0.001. Data are presented as mean ± SEM.

in warm sensation (Song et al., 2018; Tan and McNaughton, 2016; Yarmolinsky et al., 2016). However, the expression patterns of thermosensitive TRP channels in the dorsal root ganglia (DRG) are complex, and it is clear that ion channels with opposite thermal preference (hot and cold) are co-expressed in single cells (Takashima et al., 2007; Vandewauw et al., 2018). The complexity of the expression of *trp* channels and thermal response properties of peripheral sensory afferents prompted us to ask whether patterned sensory input or labeled sensory-afferent lines for temperature drive warm or cool perception.

RESULTS

Warm Perception in Mice

We used a goal-directed thermal perception task for head-restrained mice (Milenkovic et al., 2014). The glabrous skin of the right forepaw of water-restricted mice was tethered to a Peltier element (Figure 1A). The Peltier element was held at a baseline temperature of 32°C, and brief warming stimuli of 10°C (total duration 4 s) were applied randomly (Figure 1B). Mice were rewarded with water if they licked the sensor between stimulus onset and the re-cooling phase. If mice licked within 2 s before stimulus onset, a 3- to 30-s delay was imposed as a timeout to promote stimulus-lick association. To assess whether licking was selective to the thermal stimulus, "catch" trials were used where no warming or water reward were delivered. We then compared hit and false-alarm rates to assess learning in the task (Figure 1B). First, we used a small Peltier element (3 × 3 mm) to stimulate the center of the right forepaw; mice report cooling of this skin area within two training sessions (Milenkovic et al., 2014). However, mice given a warming stimulus to the same area exhibited similar hit and false-alarm rates, even after 14 days of training (n = 7 mice; Figure S1A). In contrast, when a larger skin area was stimulated (Peltier surface 8 × 8 mm, covering most of the forepaw glabrous skin), mice learned to report warming within three to four sessions (n = 12 mice; Figures 1C and 1D). Therefore, as in humans (Stevens et al.,

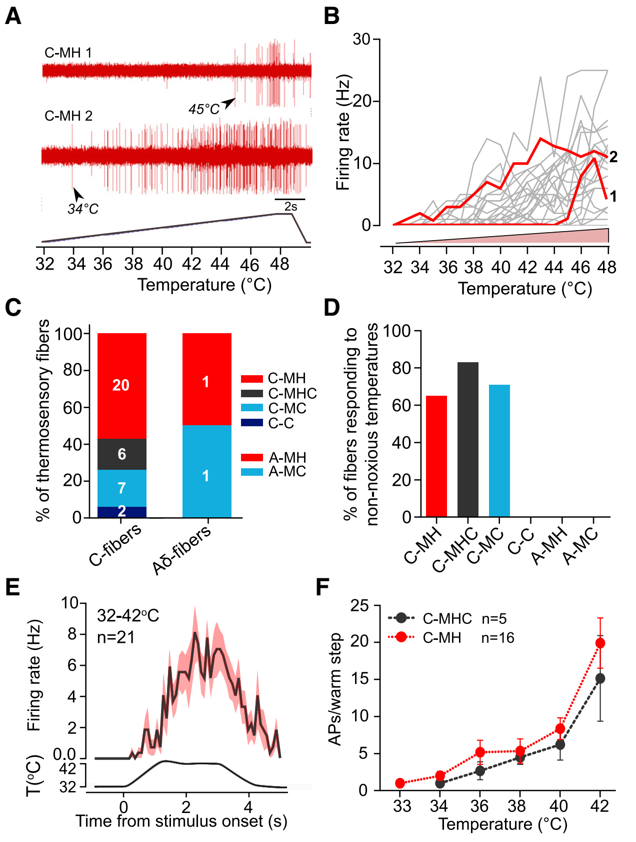

Figure 2. Forepaw Warming Evokes Spiking Responses in Polymodal C-Fibers

(A) Example of two C-MH fibers firing during a 1°C/s heat ramp (one low and one high threshold).

(B) Firing rates of all heat-responsive fibers during 1°C/s heat ramp (gray lines). Example traces from (A) are shown in red.

(C) Proportions of thermosensory C-fibers and A-fibers. C-MH, C-mechanoheat; C-MHC, C-mechanoheatcold; C-MC, C-mechanocold; C-C, C-cold; A-MH, A-mechanoheat; A-MC, A-mechanoheatcold.

(D) Percentage of fibers-in-class responsive to non-noxious warming (<42°C) and/or cooling (>22°C).

(E) PSTH of mean spike rate of all heat-responsive fibers during 42°C heat ramps.

(F) Mean number of action potentials per warm step of C-MH and C-MHC fibers did not differ (repeated-measures two-way ANOVA with Bonferroni post hoc analysis).

Data are presented as mean ± SEM.

1974), spatial summation is critical for warmth perception. Next, we measured perceptual thresholds for warming by reducing stimulus amplitude after mice had learned to report a 10°C stimulus. Mice were able to report a warming stimulus of just 1°C (from 32°C to 33°C; Figure 1E). Thus, mice have similar perceptual thresholds for warm as humans (Frenzel et al., 2012; Stevens and Choo, 1998).

Mice Report Forepaw Warming with Lower Fidelity than Cooling

We next compared the perceptual performance of mice to warm and cool stimuli delivered with the larger 8 × 8 mm Peltier from 32°C baseline. Mice learned the cooling task much more rapidly than the warming task; for example, for cooling, hit and false-alarm rates were already significantly different after the first training session (n = 7 mice, p < 0.0001; Figures S1D and S1E). To more directly compare performance in the warming and cooling detection task, we used d′ measurements (sensitivity index; see STAR Methods) and found that cooling-trained mice had higher d′ values than warming-trained mice throughout all training sessions (Figure S1E). Moreover, we found that mice were able to report a cooling stimulus of just 0.5°C (Figure S1F), whereas warm-trained mice were not (Figure S1E). Thus, as in humans, cooling perception has a lower threshold than for warming (Frenzel et al., 2012; Stevens and Choo, 1998).

In warm- and cool-trained mice, peri-stimulus time histograms (PSTHs) of the lick latencies showed that first lick responses to cooling peaked within the first second of stimulation; however, the timing of first licks to warm were more variable (Figures S1G–S1I). Warm-trained mice reported the stimulus with a mean latency of 0.87 ± 0.07 s compared to just 0.31 ± 0.03 s for cool-trained mice (n = 12 warm-trained mice, n = 7 cool trained mice; data from the training session with shortest mean latency among sessions with d′ >1.5, p < 0.0001; Figures S1I and S4E). Consistently longer latencies for warm compared to cool were observed in all training sessions (Figures S1J and S1K). Overall, these data indicate that mice sense warm with poorer spatial and temporal resolution than for cool.

Mice Discriminate between Non-noxious Warming and Cooling

To investigate whether mice are able to discriminate warming from cooling, we inserted randomly timed cooling stimuli into a warm stimulus detection session (Figure S2A). Warm-trained mice did not lick in response to cooling, indicating that mice correctly discriminate cooling from warming. Interestingly, warm-trained mice licked during the warming phase of the inserted cooling stimulus (n = 7 mice; Figure S2B). Similarly, we inserted warm stimuli into cool detection sessions (n = 7 mice; Figure S2C). Cool-trained mice withheld licking to the inserted warm stimulus and only responded during the cooling phases of the warm stimulus (Figure S2D). Thus, in this task, mice learn to report the direction of temperature change rather than its absolute value.

Polymodal C-Fibers Are Activated by Non-noxious Warm and Cool

We next asked which populations of cutaneous sensory neurons convey perceptually relevant warming information to the CNS. Using an *ex vivo* skin-nerve preparation of the medial and ulnar nerves innervating the glabrous skin of the forepaw (Walcher et al., 2018), we recorded from temperature-sensitive single fibers using a 1°C/s heating or cooling ramp (warming, 32°C to 48°C; cooling, 32°C to 12°C). We surveyed all types of fibers and characterized in detail those with thermally driven activity. All warm-driven fibers increased their firing rate monotonically as temperature increased (Figures 2A and 2B). This was also true of C-fibers innervating hindpaw glabrous skin (n = 152 fibers tested; data not shown). The majority of thermally driven afferents could be classified as polymodal C-fibers. These polymodal

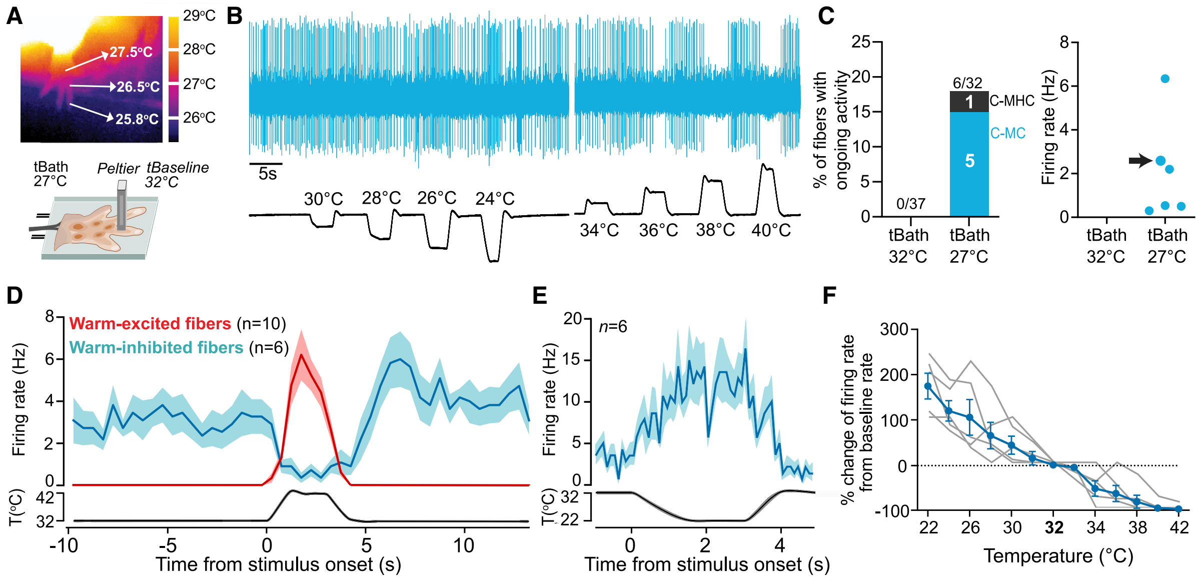

Figure 3. Warm-Inhibited C-Fibers with Ongoing Activity at Physiological Skin Temperatures

(A) Top: thermal image of the mouse forepaw at room temperature, with a paw temperature of 26°C–28°C. Bottom: schematic of forepaw afferent recordings using the *ex vivo* skin-nerve preparation bath temperature set to 27°C.

(B) Example of a C-MC fiber with ongoing activity. Cool ramps increased spike rate and warm ramps silenced spike activity.

(C) Proportion of C-fibers with ongoing activity found at 32°C and 27°C.

(D) PSTH spike rate of warm-excited fibers and warm-inhibited fibers during 10°C warm ramp.

(E) PSTH of spike rate of all warm-inhibited units during 10°C cool ramp.

(F) Percentage firing rate change in C-fibers with ongoing activity (gray lines) and mean activity change (blue, from ongoing firing rate) to cool and warm. Data are presented as mean ± SEM.

afferents had conduction velocities below 1.2 ms^{-1}, showed little or no ongoing firing, and were robustly activated by mechanical stimuli. C-fibers can simply be classified according to the types of stimulus modalities that activate them (Fleischer et al., 1983; Lewin and Mendell, 1994); thus, fibers responding only to mechanical and heat stimuli are termed C-mechanoheat (C-MH; 20/37), to mechanical heat and cold C-mechanoheatcold (C-MHC; 6/37), or mechanical and cold stimuli C-mechanocold (C-MC; 7/37) (Figure 2C). Only two fibers without a mechanosensitive receptive field were found and classified as C-cold fibers (C-C; 2/37), and a further two afferents (2/9) with Aδ-fiber conduction velocities (1.2–10 ms^{-1}) were found to be temperature sensitive and classified as A-MH (n = 1) and A-MC (n = 1) (Figure 2C). The majority of polymodal C-fibers (C-MH, C-MC, and C-MHC) responded to non-noxious temperatures, defined as spiking to stimuli below 42°C for warming or above 22°C for cooling (Figure 2D). None of the C-C or Aδ-fibers responded to non-noxious temperatures (Figure 2D).

We stimulated thermosensitive C-fibers with a series of 4 s warming and cooling stimuli with the same temporal features used in behavioral experiments (Figure 2E). PSTHs of spike latency during 32°C–42°C warm stimuli demonstrated that sparse warm-evoked spiking is observed within the first few hundred milliseconds after stimulus onset, but firing activity peaked later (Figure 2F). The mean C-fiber spike rate increased with increasing warm step amplitude (Figure 2F). Two warm-sensitive C-MH fibers were found to be activated by a 1°C warm step (32°C–33°C), the smallest warm step reliably detected by the mouse (Figure 1E). Firing was sparse with such small stimuli, consistent with the need for spatial summation to detect warm (Figure S1A).

Ongoing Activity of Cool-Sensitive C-Fibers at Physiological Skin Temperatures

Like previous studies on rodent nociceptors (Koltzenburg et al., 1997; Lynn and Carpenter, 1982; Zimmermann et al., 2009), we made *ex vivo* skin-nerve recordings with a bath temperature of 32°C. We had assumed that paw skin temperature in the mouse is 32°C; however, thermal imaging of awake mice revealed that forepaw skin temperature is between 26°C and 28°C (Figure 3A). To mimic the skin temperature during behavior, we re-investigated the thermosensory profile of forepaw afferents with the bath temperature maintained at 27°C (Figure 3A) but with the same Peltier baseline (32°C) and temperature steps as before. Again, most heat- and cool-responsive units were polymodal C-fibers (Figure S3A). Intriguingly, we observed a new population of C-fibers with ongoing spike activity in the absence of externally applied thermal stimuli (Figure 3B). These fibers are reminiscent of low-threshold cold receptors in the cornea (Belmonte et al., 2009) and may correspond to recently described menthol-sensitive Vglut3^{lineage} sensory neurons described *in vitro* with ongoing activity (Griffith et al., 2019). The physiological properties of these fibers closely resembled thermally responsive units recorded in humans (Campero and Bostock, 2010; Campero et al., 2001), monkeys (Dubner et al., 1975), and rabbits (Shea

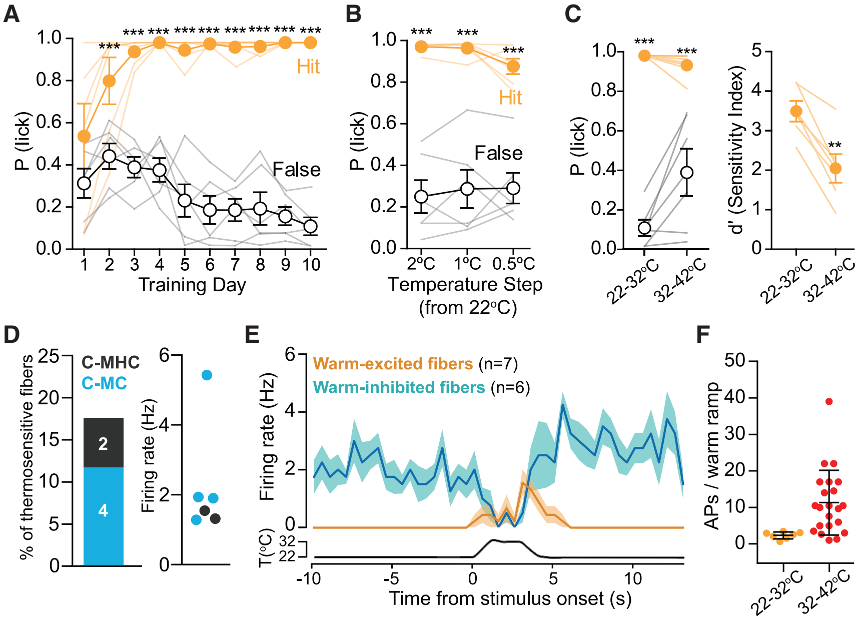

Figure 4. Warm Perception from 22°C Baseline and Its Afferent Coding

(A) Learning curve of mice trained to report a 22°C to 32°C warming step. Mice reliably report the stimulus from the second session on (n = 6 mice, two-way repeated-measures ANOVA with Bonferroni post hoc tests).

(B) Mice detect a warming step of 0.5°C starting from a baseline of 22°C (n = 6 mice, two-way repeated-measures ANOVA with Bonferroni post hoc tests).

(C) Left: the same mice reliably detect warm from 32°C or 22°C baseline; hit and false-alarm rate differences were statistically significant (n = 6 mice, two-way repeated-measures ANOVA with Bonferroni post hoc tests). Right: the sensitivity index (d′) was poorer for warming steps from a 32°C baseline compared to 22°C (n = 6 mice, p = 0.0014, paired t test).

(D) The proportion of cool-fibers with ongoing activity (left) and mean their firing rates (right) recorded at 22°C.

(E) PSTHs of warm-inhibited fibers and warm-excited fibers during 22°C–32°C stimuli.

(F) Average spike count of all warm-excited fibers during 22°C–32°C and 32°C–42°C stimuli.

*p < 0.05, **p < 0.01, and ***p < 0.001. Data are presented as mean ± SEM.

and Perl, 1985). C-fibers with ongoing activity at 27°C made up 19% of all thermosensitive fibers recorded and were further characterized as polymodal C-fibers (5 C-MCs and 1 C-MHC). Fibers with ongoing activity displayed firing rates at 27°C between 0.2 and 6 Hz and increased firing to cooling and decreased firing to warming (Figure 3C). We plotted PSTHs of C-fiber firing to the 32°C–42°C warm ramp used for behavioral training. Warm stimuli activated a separate population of polymodal C-fibers with a time course that mirrored the inhibition of cool-sensitive fibers with ongoing activity (Figure 3D). Cooling ramps from 32°C to 22°C evoked robust firing in a larger population of polymodal C-fibers (C-MCs and C-MHCs; Figure S3G), which included all fibers with ongoing activity at 27°C (Figure 3E). Using small step changes in thermal ramps (illustrated in Figure 3B), we probed how firing rates changed with temperature in cool-sensitive fibers with ongoing activity. As expected, these C-fibers increased their firing rates with cooling and were progressively silenced by warming (Figure 3F).

C-fibers with monotonically increasing firing rates to increasing temperature represented the majority of thermosensitive afferents (Figures S3D–S3F). However, we also observed small populations of cool-responsive fibers and warm-responsive afferent fibers that only responded to specific ranges of temperatures and were inhibited by noxious temperatures (Figures S3D–S3F). Warm-preferring units that stopped firing at noxious heat temperatures during the 1°C/s heat ramp were only found in experiments where the skin was maintained at 27°C and not at 32°C, while cool-preferring units were found in both sets of experiments (Figure S3F).

Warm-Inhibited C-Fibers Are Key Drivers of Warm Perception

We next examined warm perception at lower baseline temperatures. We trained mice at a baseline of 22°C to report a 10°C warm step (22°C–32°C). Mice quickly learned the task (n = 6 mice, p < 0.0001 since training session 2; Figures 4A and S4A) and had a detection threshold of just 0.5°C (Figure 4B). In the same mice, we then shifted the baseline to 32°C and delivered 10°C steps. Detection of 10°C warm steps from 22°C baseline was more robust than from 32°C baseline (n = 6 mice, p < 0.005, mean d′ = 3.43 ± 0.26 versus 2.05 ± 0.36; Figure 4C). Mice trained to report warm of 22°C–32°C displayed faster detection latencies than those trained at 32°C–42°C (n = 6 and n = 12 mice respectively, p < 0.05, 0.59 ± 0.04 s versus 0.87 ± 0.07 s; Figures S4D–S4F). In addition, mice reported a 10°C cooling step from a 22°C baseline (n = 6, p < 0.0001 from session 1; Figures S4B–S4D), but here, response latency increased from 0.31 ± 0.03 s in mice trained to report 32°C to 22°C (n = 7 mice) to 0.75 ± 0.06 s in mice trained to report 22°C to 12°C (n = 6 mice) (p < 0.0001; Figures S4E and S3F). These data indicate that warm perception is more acute at lower baseline temperature values.

Next, we compared perceptual performance with afferent responses with a bath temperature 27°C to mimic paw temperature and a Peltier baseline of 22°C. Here, we found cool-sensitive fibers with ongoing spiking rates similar to those found with a bath temperature of 27°C (Figure 4D), which were silenced by a 22°C–32°C warming step (Figure 4E). We also recorded cool-excited fibers that increased their firing rates to a 22°C to 12°C cold stimulus (Figure S4H). Interestingly, we also observed warm excited C-MH and C-MHC fibers, but these fibers were only sparsely and weakly activated compared to when warming stimuli were given from a starting temperature of 32°C (Figures 4E and 4F). Thus, from a 22°C baseline, mice show robust warmth perception, despite a substantial reduction in the strength of excitatory drive from warm-excited afferents.

CellPress

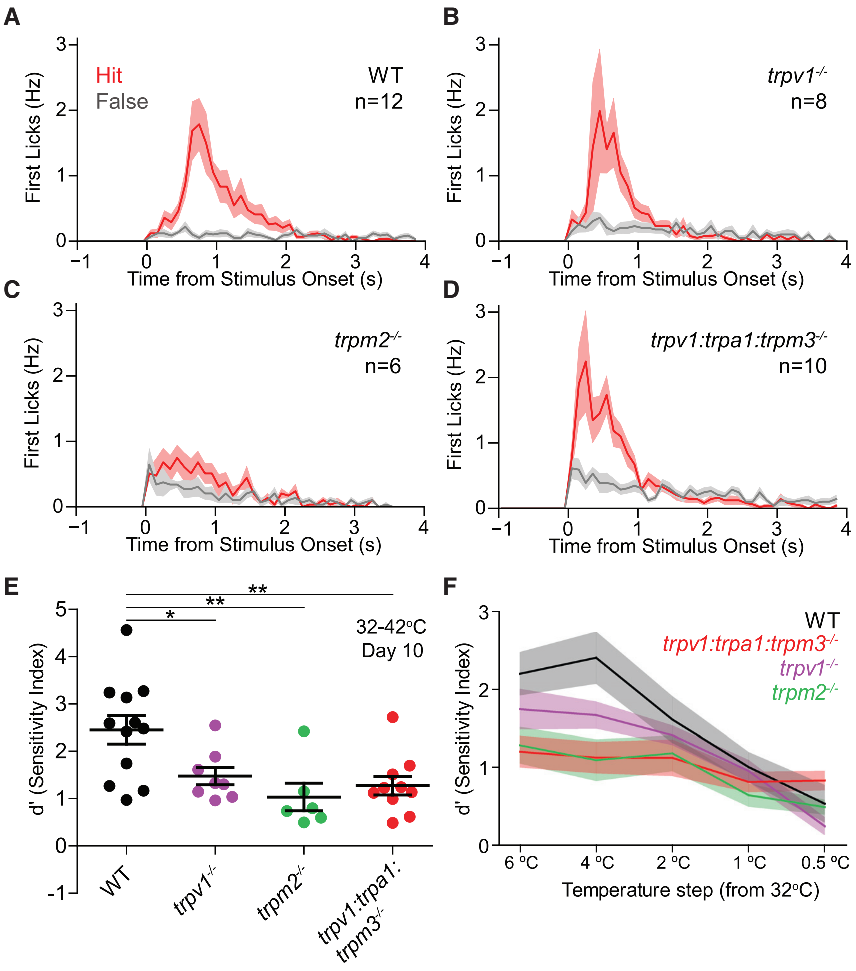

Figure 5. TRPV1, TRPM2, TRPA1, and TRPM3 Are Not Absolutely Required for Warm Perception

(A) Lick PSTH warm-trained control WT mice at day 10 showing distribution of first licks to the warm stimulus (red) or during catch trials (gray).

(B) Same as (A), but for *trpv1*$^{-/-}$.

(C) Same as (A), but for *trpm2*$^{-/-}$; note the small difference between hit and false-alarm lick rates.

(D) Same as (A), but for *trpv1:trpa1:trpm3*$^{-/-}$.

(E) Sensitivity (d′) analysis revealed all *trp* mutant mice detect warm better than chance (d′ = 0). However, all *trp* mouse mutants had partial perceptual deficits compared to WT mice (WT mean d′ = 2.45 ± 0.30, *trpv1*$^{-/-}$ d′ = 1.48 ± 0.19 versus WT, p < 0.05; *trpm2*$^{-/-}$ d′ = 1.03 ± 0.29 versus WT p < 0.01, *trpv1:trpa1:trpm3*$^{-/-}$ d′ = 1.28 ± 0.20 versus WT; p < 0.01 unpaired t tests).

(F) Sensitivity (d′) values of WT mice and *trpv1*$^{-/-}$, *trpm2*$^{-/-}$, and *trpv1:trpa1:trpm3*$^{-/-}$ mice during warm threshold sessions.

*p < 0.05 and **p < 0.01, Data are presented as mean ± SEM.

Warm-Responsive TRP Channels Are Not Absolutely Required for Warmth Perception

A number of TRP channels are thought to be required for warm detection. We therefore used mice with targeted *trp* channel gene deletions to ask which channels are required for the sensory coding of warm perception. We trained mutant (backcrossed onto C57BL/6 background) and wild-type (WT) C57BL/6 mice to report a 10°C warm stimulus (from 32°C baseline) using the (8 × 8 mm) Peltier device. We found that *trpv1*$^{-/-}$ mice learned to report non-painful warm stimulation of the forepaw (32°C–42°C) (n = 8 mice; Figures 5B and S5A). Performance (Figures 5E, 5F, and S5G) and lick-response latencies (Figures 5A and 5B) were similar to WT (Figures 5A and 5B). Like WT mice, *trpv1*$^{-/-}$ mice could detect a temperature change of 1°C (32°C–33°C; Figure S5D); thus, TRPV1 appears to be dispensable for warm perception.

trpm2$^{-/-}$ mice were also able to learn to report non-painful warm (32°C–42°C) over the 10-day training period (n = 6 mice; Figures 5E, S5B, and S5G). However, we found that learning performance was impaired in *trpm2*$^{-/-}$ compared to WT mice (Figures 5E, 5F, and S5G). Additionally, lick PSTHs of *trpm2*$^{-/-}$ mice suggested poorer detection of the stimulus (Figure 5C). Moreover, *trpm2*$^{-/-}$ mice had slightly higher warm perceptual thresholds (2°C) than WT mice (1°C) (Figure S5E). These data indicate that, while TRPM2 plays a role in warm perception, it is not essential.

Finally, we trained mice in which the genes encoding the TRPV1, TRPA1, and TRPM3 ion channels were ablated (*trpv1:trpa1:trpm3*$^{-/-}$). These mice are unable to sense acute noxious heat (Vandewauw et al., 2018), but many C-fibers that encode noxious heat are also activated by non-noxious warm (Figure 2B). Surprisingly, *trpv1:trpa1:trpm3*$^{-/-}$ mice learned to report warming stimuli of 32°C–42°C (n = 10; Figures 5D, 5E, S5C, and S5G) and could also sense small amplitude warming stimuli (Figures 5F and S5F). In addition, *trpv1:trpa1:trpm3*$^{-/-}$ mice could sense warming stimuli of 22°C–32°C (n = 10; Figures S5K and S5L) as well as cooling stimuli of 32°C to 22°C (n = 6; Figures S5H–S5J). Together, these findings reveal that mice perceive warm in the absence of TRPV1, TRPM2, TRPM3, and TRPA1.

Recordings from hindpaw C-fibers in *trpv1*$^{-/-}$, *trpm2*$^{-/-}$, and *trpv1:trpa1:trpm3*$^{-/-}$ mutant mice (from a 32°C baseline) indicated that the ability of polymodal C-fibers to detect both warm and cooling stimuli was largely unchanged compared to WT mice (Figures S6D and S6F). The only significant differences noted was that the proportion of cool-sensitive C-fibers (C-MHC and C-MC fibers) was significantly reduced in *trpm2*$^{-/-}$ mice compared to controls (Figure S6A). Additionally, C-MH and C-MHC fibers recorded from *trpv1*$^{-/-}$ mice were normally activated by non-noxious temperatures but in contrast to WT polymodal nociceptors failed to dramatically increase their firing rates when stimulated into the noxious range (>44°C) (Figures S6B–S6E).

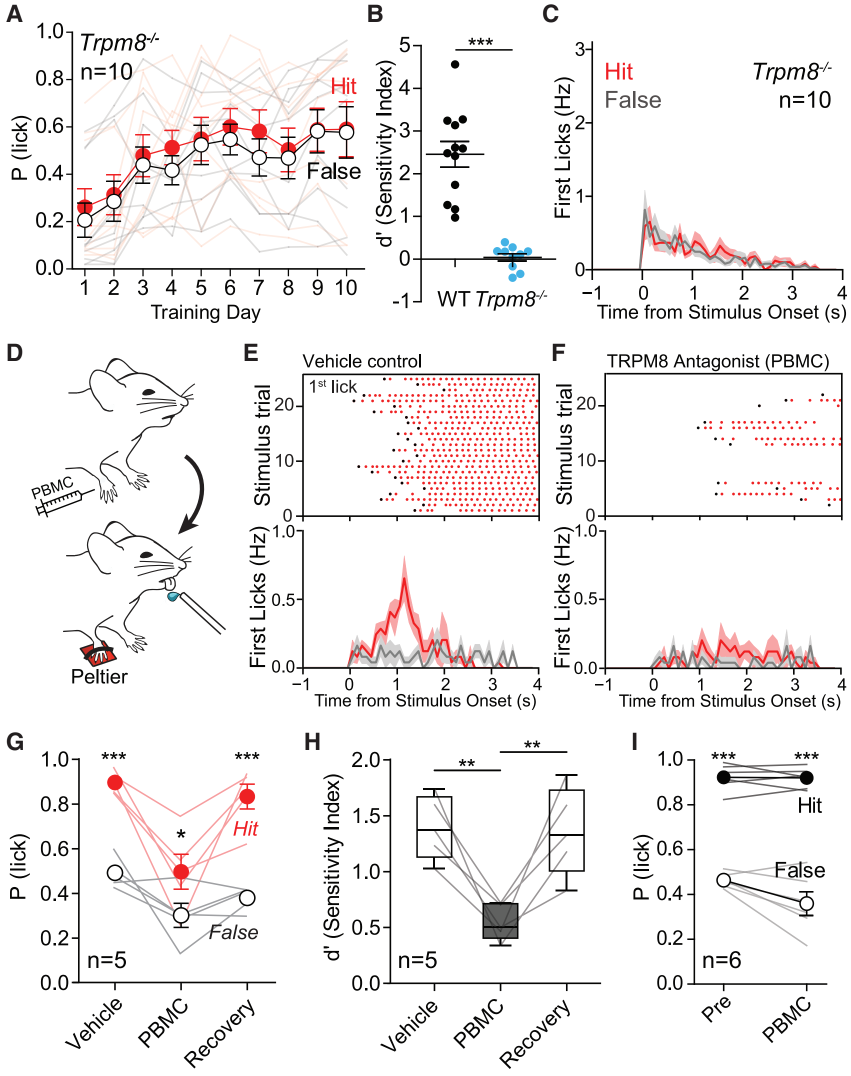

Figure 6. TRPM8 Is Required for Warm Perception

For a Figure360 author presentation of this figure, see https://doi.org/10.1016/j.neuron.2020.02.035. Figure360▷

(A) *trpm8*$^{-/-}$ mice showed no warm detection, as hit and false-alarm rates were the same throughout training (n = 10; two-way repeated-measures ANOVA with Bonferroni post hoc tests).

(B) After 10 training days, *trpm8*$^{-/-}$ mice had d′ values ~ 0 (chance performance), which was significantly different compared to WT (WT mean d′ = 2.45 ± 0.30 and n = 12, *trpm8*$^{-/-}$ d′ = 0.04 ± 0.09 and n = 10; p < 0.0001 versus WT, unpaired t test).

(C) PSTH of the first licks of *trpm8*$^{-/-}$ mice at day 10. No difference between presence (red) and absence (gray) of stimulus.

(D) Schematic representation of pharmacological experiment using the TRPM8 antagonist PBMC.

(E) Raster plot (top) from a DMSO-vehicle-treated mouse and population mean first-lick latency PSTH (bottom).

(F) Raster plot (top) from a PBMC-treated mouse and population mean first-lick latency PSTH (bottom) show much reduced warm detection.

(G) Hit and false-alarm rate differences was reversibly reduced in PBMC-treated mice compared to vehicle, with recovery 24 h after treatment (n = 5, two-way ANOVA with Bonferroni post hoc analysis).

(H) Sensitivity (d′) indices were reversibly impaired in PBMC-treated mice compared to vehicle controls (n = 5, paired t tests between PBMC and DMSO or recovery groups).

(I) PBMC-treated mice report tactile stimuli normally (n = 6, two-way ANOVA with Bonferroni post hoc analysis).

*p < 0.05, **p < 0.01, and ***p < 0.001. Data are presented as mean ± SEM.

Warm Perception Requires TRPM8 Channels

C-fibers with ongoing cool-driven activity may be dependent on the cold-activated channel TRPM8, and this prompted us ask if *trpm8*$^{-/-}$ mice can learn to detect warm. We trained *trpm8*$^{-/-}$ mice on our warm task (32°C–42°C) for 10 days, and they completely failed to report warm (n = 10 mice; Figures 6A and 6B). False-alarm lick rates remained similar to hit rates over the training session; licking was poorly correlated to the stimulus time, and d′ measurements were significantly reduced compared to WT mice trained for the same number of sessions (Figures 6B, 6C, and S5G). However, *trpm8*$^{-/-}$ mice easily learned to report mechanical stimuli applied to the forepaw (n = 5 mice, p < 0.001 session 1; Figures S5M and S5N) and auditory stimuli (data not shown) with short lick latencies, demonstrating that the warm perception deficit was not due to a general learning impairment. *trpm8*$^{-/-}$ mice were also unable to report cooling (32°C to 22°C) when delivered via a larger, 8 × 8 mm Peltier (data not shown); previous data were obtained using a smaller stimulus area of 3 × 3 mm (Milenkovic et al., 2014). Thus, unexpectedly, TRPM8 expression appears to be required for warm sensation in mice.

The loss of warm sensation in *trpm8*$^{-/-}$ mice could be an indirect consequence of the early developmental loss of cool information reaching the brain. We addressed this issue by acutely inactivating TRPM8 in the forepaw of WT mice using PBMC (1-Phenylethyl-(2-aminoethyl)[4-(benzyloxy)-3-methoxybenzyl]carbamate), a selective antagonist of TRPM8 that has been shown to suppress cooling-responsive cells and reduce cooling-evoked behavioral responses in mice (González et al., 2017; Griffith et al., 2019; Knowlton et al., 2013; Yudin et al., 2016). We first trained WT animals to report warm stimuli and then we pharmacologically inactivated TRPM8 by performing a transdermal injection in the plantar side of the right forepaw (Figures 6D–6F). Twenty minutes after PBMC application, mice showed a significantly poorer warm detection performance compared to DMSO-treated controls as shown by reduced d′ indices (n = 5 mice, p < 0.01, mean d′ 1.39 ± 0.13 vehicle injected versus 0.55 ± 0.07 PBMC injected; Figures 6G and 6H). Furthermore,

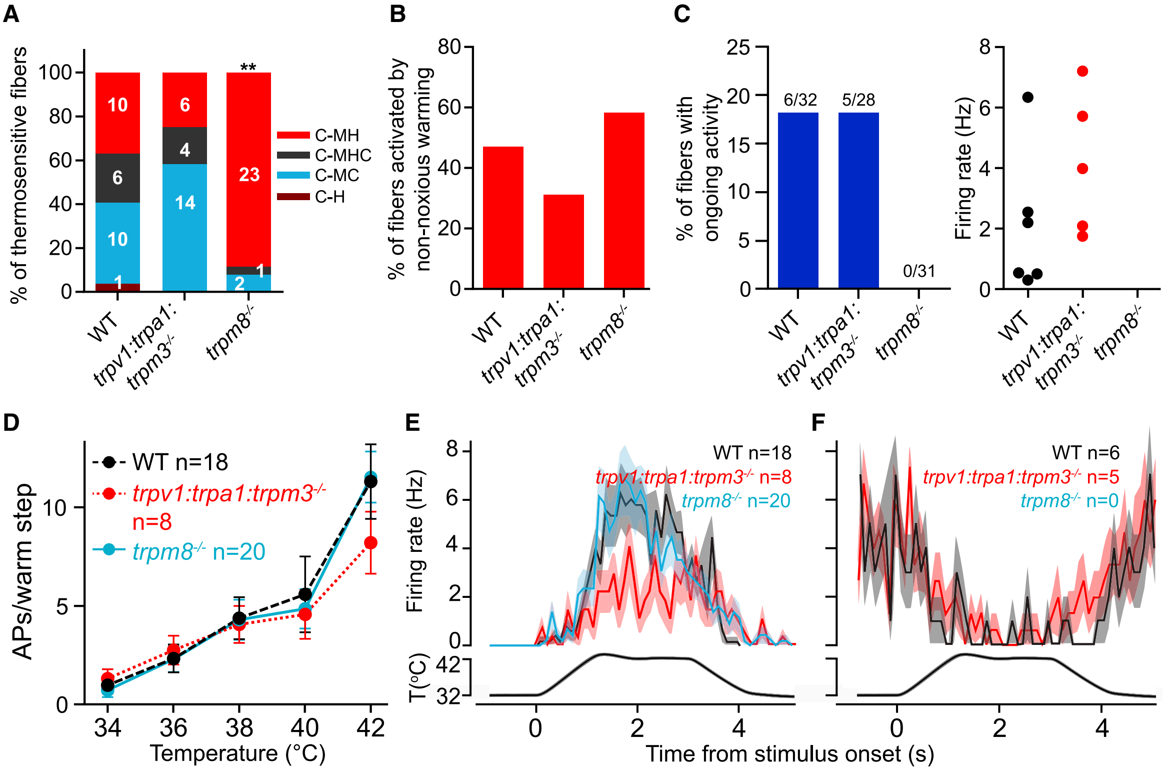

Figure 7. *trpm8*$^{-/-}$ Mice Lack Warm-Evoked Silencing of C-Fibers

(A) Proportions of thermosensitive forepaw C-fibers were not significantly different between WT and *trpv1:trpa1:trpm3*$^{-/-}$ mice, but there was dramatic reduction in cold-sensitive C-MC and C-MHC fibers in *trpm8*$^{-/-}$ mice compared to WT.

(B) Proportions of warm-responsive fibers did not differ between control and *trpv1:trpa1:trpm3*$^{-/-}$ and *trpm8*$^{-/-}$ mice.

(C) Absence of cool-driven C-fibers with ongoing activity in *trpm8*$^{-/-}$ mice. The incidence and firing rates of cool-driven C-fibers with ongoing activity was not different between *trpv1:trpa1:trpm3*$^{-/-}$ mice and WT controls.

(D) Mean warm-evoked firing rates did not differ among control, *trpv1:trpa1:trpm3*$^{-/-}$, and *trpm8*$^{-/-}$ mice (repeated-measures two-way ANOVA with Bonferroni post hoc analysis).

(E) PSTHs of mean spike rates to a warm ramp recorded from warm-activated C-fibers showed comparable responses between genotypes.

(F) PSTHs from warm-inhibited, cool-driven fibers (not present in *trpm8*$^{-/-}$ mice).

Data are presented as mean ± SEM.

the latencies to report the stimuli in the successful hit trials were longer when mice were given local PBMC (n = 5 mice, $p < 0.001$, mean latency 1.37 ± 0.05 s vehicle injected versus 2.02 ± 0.07 s PBMC injected; data not shown). These effects were reversible, as mice showed baseline levels of performance and latencies 24 h after PBMC injection (Figures 6G and 6H). Moreover, the effects of PBMC injection were restricted to thermal perception, as transdermal PBMC injections in mice trained to report a tactile stimulus had no effect on this behavior (Figure 6I). Together, these data suggest that functional TRPM8 channels expressed in the forepaw are acutely required for warm perception.

Warm-Inhibited C-Fibers Are Absent in *trpm8*$^{-/-}$ Mice

The presence of warm perception in WT and *trpv1:trpa1:trpm3*$^{-/-}$ and absence in *trpm8*$^{-/-}$ mice prompted us to examine forepaw afferent responses from these two strains. Interestingly, *trpv1:trpa1:trpm3*$^{-/-}$ mice cannot detect noxious heat, a modality signaled by the same polymodal C-fibers that respond to warm. We therefore made forepaw afferent recordings from WT, *trpv1:trpa1:trpm3*$^{-/-}$, and *trpm8*$^{-/-}$ mutant mice with the bath temperature set to 27°C. The proportions of thermosensory fiber subtypes were comparable between WT and *trpv1:trpa1:trpm3*$^{-/-}$ mice, but *trpm8*$^{-/-}$ mice showed an expected loss of cool-sensitive fibers (Figure 7A). Notably, we did not find any cool-responsive fibers in *trpm8*$^{-/-}$ mice with ongoing activity (n = 7 mice; Figure 7C), presumably due to the dramatic reduction in C-fiber cool sensitivity. In contrast, active cool fibers with ongoing activity were present in *trpv1:trpa1:trpm3*$^{-/-}$ mice and had firing rates similar to those found in WT mice (Figure 7C).

Both noxious heat- (above 42°C) and warm-excited (32°C–42°C) fibers were present in *trpm8*$^{-/-}$ and *trpv1:trpa1:trpm3*$^{-/-}$ mice with similar proportions to WT mice and were all polymodal (Figures 7B–7E). Forepaw C-fibers with monotonic spiking responses to warm were observed in both *trpm8*$^{-/-}$ and *trpv1:trpa1:trpm3*$^{-/-}$ mice (Figures 7D and 7E), but, as previously reported (Vandewauw et al., 2018), there was a significant reduction in noxious heat responses at 48°C in *trpv1:trpa1:trpm3*$^{-/-}$ mice (Figure S7D). There were no significant differences in heat-evoked spike activity in C-fibers between control and *trpm8*$^{-/-}$ mice

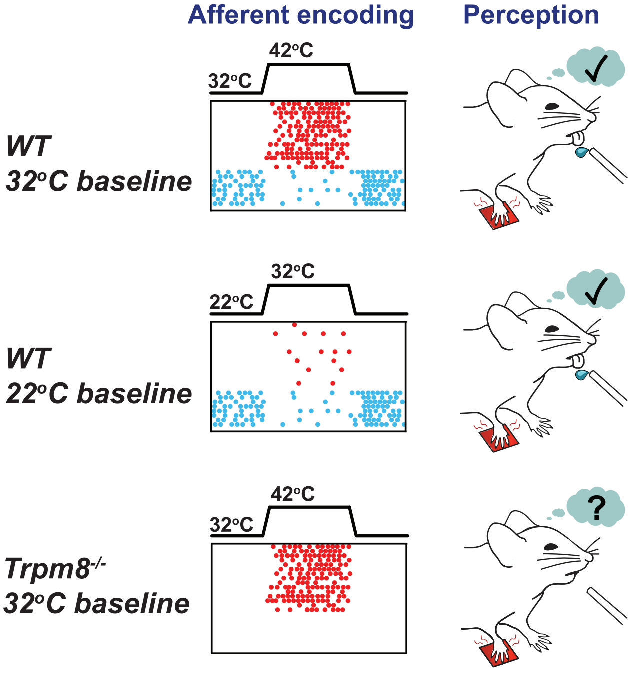

Figure 8. Model of Afferent Encoding of Perceived Warmth

Forepaw warming recruits two populations of sensory afferents: (1) activation of warm-sensitive C-fibers that are silent at rest (red) and (2) decreased spiking in a subset of cool-sensitive C-fibers that are active at rest (blue). A warm step from 32°C to 42°C elicits both types of responses, and a warm step of 22°C to 32°C evokes mainly warming-evoked inhibition. In the absence warm-evoked inhibition of C-fibers with cool-driven ongoing activity, warm detection fails (*trpm8*$^{-/-}$ mice), even in the presence of warm-evoked firing (red).

(Figures 7D and S7D). Cool-preferring and monotonic cold fibers were also present in both *trpm8*$^{-/-}$ mice and *trpv1:trpa1:trpm3*$^{-/-}$ mice (Figure S7C). We conclude that for warm perception, input from warm-inhibited cool-sensitive C-fibers is necessary. On the other hand, input from warm-excited C-fibers alone appears insufficient to allow mice to perceive warm.

DISCUSSION

While the afferent neurons and ion channels necessary for cool perception have been studied extensively (Bubb et al., 1994; Dhaka et al., 2007, 2008; Knowlton et al., 2013; McKemy et al., 2002; Milenkovic et al., 2014; Pogorzala et al., 2013), far less is known about non-noxious warm perception (Bokiniec et al., 2018; Filingeri, 2016). Here, we show that mice have similar perceptual thresholds for warm as humans. We identify C-fibers (C-MHC and C-MC fibers) that show cool-driven ongoing activity at physiological temperatures but are inhibited by warming stimuli as critical players in warm sensation. The activity of these warm-inhibited fibers is dependent on TRPM8 channels, as these fibers are absent in *trpm8*$^{-/-}$ mice that cannot detect warm (Figure 7). The second population of polymodal C-fibers (C-MHC and C-MH fibers) was sparsely activated by warm stimuli. None of the thermo-*trp* channel knockout mice examined (*trpv1*$^{-/-}$, *trpm2*$^{-/-}$, and *trpv1:trpa1:trpm3*$^{-/-}$) exhibited complete loss of warm coding by polymodal C-fibers (C-MHC and C-MH fibers), and all mutant mice could detect warm (Figure 5). We propose that it is the concurrent inhibition and excitation of these two polymodal channels that provide the sensory code for warm perception (Figure 8).

Non-painful Warm and Cool Perception Is Similar in Mouse and Human

We show that mice exhibit remarkably similar warm and cool perceptual abilities to humans. Mice detect skin warming of just 0.5°C and skin cooling of 0.5°C from a 32°C or 22°C baseline, values that closely match forearm thermal thresholds in humans (Stevens and Choo, 1998). As in humans, the ability of mice to report forepaw warming is strongly dependent on spatial summation (Figure S1A) (Filingeri, 2016; Stevens and Choo, 1998; Stevens et al., 1974), and mice easily discriminate non-noxious warming from cooling stimuli. Mice show higher sensitivity to cool than to warm (Figure 1). In addition, mice reported warm steps slightly better when the baseline was 22°C, a task that might rely more on inhibition of cool fibers, than at 32°C (Figure 7). In humans the perception of skin cooling is more acute and reliable than for warm (Stevens and Choo, 1998). The similarity in thermal perceptual ability between mice and humans suggests that both sensory coding and central processing of temperature discrimination has a common neural basis.

No Labeled Line for Warm Sensation

We did not record any forepaw C-fibers that might form a labeled afferent line tuned exclusively to warm. In mice, warm-sensitive afferents recorded at a baseline skin temperature of 32°C all responded monotonically to increasing skin temperature and also responded to high-threshold mechanical stimuli (Figure 2). The mouse forepaw has a much higher surface to volume ratio than the primate hand; thus, maintenance of skin temperature close to body core temperature could be problematic in this appendage. Thermal imaging measurements revealed that the mouse forepaw temperature was lower than core body temperature at between 27°C and 29°C. This observation led us to investigate warm perception and sensory coding at these more physiological temperatures. Interestingly, at a baseline temperature of 27°C, we found a small number of warm- or cool-preferring C-fibers that decrease firing rates when temperatures become noxious (Figures S3D–S3E′). However, these warm- or cool-preferring fibers were very broadly tuned to stimulus amplitude (range of ~Δ10°C) but were not dedicated thermoreceptors, as they also respond to high threshold mechanical stimuli. In a classic paper, LaMotte and Campbell (1978) showed that sparse coding of warm by dedicated thermoreceptors in the monkey hand may account for psychophysical performance in humans. However, warm-specific receptors are very rare in human skin. In one study, just 5 out of 125 C-fibers were found to exhibit the classic features of a dedicated warming receptor (Hallin et al., 1982). Thus, the warm-preferring C-fibers identified here may be the murine equivalent of more tightly tuned, dedicated thermoreceptors identified in primates. Indeed, our data are consistent with large-scale imaging of thousands of DRG neurons to thermal stimuli that has failed to identify large populations of sensory neurons that respond to specific ranges of warm (Chisholm et al., 2018; Wang et al., 2018; Yarmolinsky et al., 2016). Importantly, we also observed no decrease in the incidence of warm-preferring

or warm-activated C-fibers in *trpm8*$^{-/-}$ mice that cannot detect warm (Figure 7). Thus, dedicated warm receptors alone cannot provide sufficient information to drive warm perception.

Sparse Coding for Warm

Using warm as a search stimulus, we found that the majority of warm-coding afferents were polymodal C-fibers: C-MH (warm-excited), C-MHC (warm-excited or warm-inhibited), or C-MC (warm-inhibited) fibers. We found that individual polymodal C-fibers are only sparsely activated (or inhibited) by warm stimuli around the perceptual threshold, with firing rates changing only slightly for the smallest warm steps (Figures 2 and 3). In rodents, most reports have shown that more than 60% of all C-fibers show polymodality, including activation by cold and heat (Milenkovic et al., 2008, 2014; Zimmermann et al., 2011). Here, using slow warming and cooling ramps, we show that the vast majority (>60%) of mouse polymodal C-fibers show changes in spiking (Figure 2D). We counted the total number of unmyelinated C-fibers in the medial and ulnar nerves from transmission electron micrographs (Figures S7F and S7G) and found that the skin areas innervated by these two nerves may have C-fiber densities of up to 176 fibers/mm^2. The extremely high skin innervation density of the forepaw has already been observed for mechanoreceptors that mediate touch sensation (Walcher et al., 2018; Wetzel et al., 2017). Based on our recordings, ~36% of all C-fibers are responsive to innocuous skin temperature change; thus, more than 60 C-fibers/mm^2 could provide some warm-related information. Only mice trained with the larger Peltier device were able to learn the warm-detection task, as mice failed to reliably learn the task with a smaller probe (Figure S1). Spatial summation of temperature information over almost the entire forepaw (Peltier contact area ~22 mm^2) therefore seems to be required for warm detection, and this would be associated with warm-evoked firing-rate changes (inhibition and excitation) in more than 1,300 C-fibers for a 10°C temperature change. Thus, individual sensory neurons provide sparse information about warm, but this may be compensated by information being carried by large numbers of fibers. Interestingly, in human skin both polymodal C-MH and C-MHC fibers with physiological properties similar to those described here are very common (>40% of total C-fibers) (Campero and Bostock, 2010; Campero et al., 1996; Van Hees and Gybels, 1981). Thus, sparse coding of warm-evoked activity by many polymodal C-fibers may be an evolutionarily conserved mechanism for warm detection.

Heat-Activated TRP Channels Are Not Required for Warm Sensing

Recent reports indicated a role for both *trpm2* and *trpv1* in warm transduction (Tan and McNaughton, 2016; Yarmolinsky et al., 2016; but see M. Mulier, I. Vandewauw, J.V., T.V., unpublished data). Here, we did not observe any warm (32°C–42°C) encoding defect in the afferents of *trpv1*$^{-/-}$ mice, but we did observe a marked reduction in spiking beyond the noxious heat threshold (>42°C). This is in good agreement with the mild behavioral deficits in reacting to noxious heat observed in these animals (Caterina et al., 2000). We found that *trpm2*$^{-/-}$ mice do have a performance deficit in warm detection (Figure 5). However, we found no significant differences in the sensitivity of polymodal C-fibers (C-MH and C-MHCs) to non-noxious warm. Indeed, the only afferent deficit observed in *trpm2*$^{-/-}$ mice was reduced numbers of cold-sensitive polymodal C-fibers (Figure S6).

The ion channel trio composed by TRPV1, TRPA1, and TRPM3 has recently been shown to play an essential role in the encoding of acute noxious heat (Vandewauw et al., 2018). The profound noxious heat deficit in these mice allowed us to ask if warm sensation is preserved in the absence of noxious heat sensation. This question was particularly interesting considering that many polymodal C-fibers can convey both warm and noxious heat information (Figure 2). Similarly to *trpv1*$^{-/-}$ mice, the heat-sensitive fibers of *trpv1:trpa1:trpm3*$^{-/-}$ mice showed much reduced spiking in the noxious heat range, as shown previously (Vandewauw et al., 2018). Nevertheless, *trpv1:trpa1:trpm3*$^{-/-}$ mice display reduced performance, but were still able to report warm (Figure 6), consistent with behavioral thermal preference assays (Vandewauw et al., 2018). We found reduced numbers of warm-activated C-fibers in *trpv1:trpa1:trpm3*$^{-/-}$ mice, but the reduction was not statistically significantly (Figure S7).

Cool-Sensitive Afferents Are Required for Warm Perception

Cool-sensitive C-fibers are predominantly TRPM8$^+$ (Bautista et al., 2007; Dhaka et al., 2008). We confirmed here that in the absence of *trpm8*, many fewer C-fibers were found that responded to cool in the 32°C to 22°C range (Bautista et al., 2007; Milenkovic et al., 2014). Unexpectedly, we observed a complete lack of warm perception in *trpm8*$^{-/-}$ mice and a strong deficit in control mice following an acute inhibition of the TRPM8 channels in the paw but no change in the properties of warm-activated fibers in *trpm8*$^{-/-}$ mice. Instead, *trpm8*$^{-/-}$ mice lacked ongoing activity of cool-sensitive fibers and therefore the mechanism of warm-evoked inhibition was disabled. Control mice robustly detect a warm step of 22°C to 32°C, a stimulus that elicited poor spiking in warm-activated neurons, but this step evoked robust inhibition of cool-sensitive C-fibers with ongoing activity. These data suggest that warm-evoked inhibition of fibers that are active at rest are necessary for the perception of warm.

Interestingly, cool-sensitive C-fibers with ongoing activity had similar firing rates during the baselines of 32°C and 22°C (Figures 5 and 7), which suggests that they adapt their discharge rate to the background temperature and are therefore specialized in encoding magnitude of change rather than absolute temperature. Similarly, cooling-sensitive fibers showed similar responses to cooling of 32°C to 22°C and 22°C to 12°C (Figures S3G and S4H). This contrasts with warm-sensitive afferents, which showed robust spiking responses to warm at 32°C to 42°C (Figure 3D) but reduced responses to a warm step of 22°C to 32°C (Figures 4E and 4F). The idea that heat-sensitive neurons encode temperature in an absolute way but cold-sensitive neurons encode magnitude of change has been previously proposed (Wang et al., 2018; Ran et al., 2016). Our results are thus compatible with the findings from these large-scale imaging studies.

Two Polymodal Sensory Channels for Warm Sensation

We propose a model whereby two sensory information channels provide the information to drive highly sensitive and accurate detection of skin warming (Figure 8). Of these two channels,

excitation of warm-excited-sensitive and inhibition of cool-sensitive polymodal C-fibers, we show that the latter is necessary for warm detection (Figures 6 and 7). While we have not identified a mouse model or experimental situation in which warm-excited polymodal C-fibers are completely absent, we observed that warm detection performance is significantly impacted in situations where only the numbers of warm-excited C-fibers are reduced (*trpv1:trpa1:trpm3*$^{-/-}$ mice; Figures 5 and 7). We therefore propose that activity in two populations of cutaneous polymodal C-fibers is required to drive warm detection, without a need for specialized thermoreceptors. This model explains why mice do not confuse warm with cool, as it is only warm that simultaneously excites one population and inhibits the second C-fiber population. Our data now challenge the field to discover where and how these two streams of sensory information are integrated in the spinal cord or brain to drive accurate and specific thermal perception.

STAR★METHODS

Detailed methods are provided in the online version of this paper and include the following:

- KEY RESOURCES TABLE
- LEAD CONTACT AND MATERIALS AVAILABILITY
- EXPERIMENTAL MODEL AND SUBJECT DETAILS
  - Animals
- METHOD DETAILS
  - Head implanting of mice for behavioral training
  - Behavioral training
  - Skin-nerve preparation and sensory afferent recordings
  - Transdermal injections in the forepaw
- QUANTIFICATION AND STATISTICAL ANALYSIS
  - Analysis of behavior
  - Analysis of skin-nerve recordings
  - Statistical tests
- DATA AND CODE AVAILABILITY

SUPPLEMENTAL INFORMATION

Supplemental Information can be found online at https://doi.org/10.1016/j.neuron.2020.02.035.

ACKNOWLEDGMENTS

This work was supported by the European Research Council (ERC-2017-ADG-789128 to G.R.L. and ERC-2015-CoG-682422 to J.F.A.P.); the European Union (3x3Dimaging 323945 to J.F.A.P.); the Deutsche Forschungsgemeinschaft (SFB 1315 to J.F.A.P. and FOR 2143 to J.F.A.P.); the Thyssen Foundation (J.F.A.P.); and the Helmholtz Society (G.R.L. and J.F.A.P.). We thank Bettina Erdmann for help with electron microscopy. We thank Valerie Bégay, Janett König, and Charlene Memler for assistance with mouse breeding, mouse import, and training.

AUTHOR CONTRIBUTIONS

Conceptualization, G.R.L., R.P.-M., J.F.A.P., and F.S.; Methodology, G.R.L., R.P.-M., A.U., J.F.A.P., F.S., and J.W.; Formal Analyses, R.P.-M., F.S., J.W., A.U., and R.E.; Software, F.R., and R.P.-M.; Investigation, R.P.-M., F.S., J.W., and R.E.; Writing – Original Draft, R.P.-M., F.S., G.R.L., and J.F.A.P.; Resources, J.V. and T.V.; Funding Acquisition, G.R.L. and J.F.A.P; Supervision, G.R.L. and J.F.A.P.

DECLARATION OF INTERESTS

The authors declare no competing interests.

Received: January 17, 2020
Revised: February 24, 2020
Accepted: February 28, 2020
Published: March 23, 2020

REFERENCES

Bautista, D.M., Siemens, J., Glazer, J.M., Tsuruda, P.R., Basbaum, A.I., Stucky, C.L., Jordt, S.-E.E., and Julius, D. (2007). The menthol receptor TRPM8 is the principal detector of environmental cold. Nature *448*, 204–208.

Belmonte, C., Brock, J.A., and Viana, F. (2009). Converting cold into pain. Exp. Brain Res. *196*, 13–30.

Blix, M. (1882). Experimentela bidrag till losning af fragan om hudnervernas specifika energi. Uppsala Lakfor. Forh. *18*, 87–102.

Bokiniec, P., Zampieri, N., Lewin, G.R., and Poulet, J.F.A. (2018). The neural circuits of thermal perception. Curr. Opin. Neurobiol. *52*, 98–106.

Bubb, M.R., Senderowicz, A.M., Sausville, E.A., Duncan, K.L., and Korn, E.D. (1994). Jasplakinolide, a cytotoxic natural product, induces actin polymerization and competitively inhibits the binding of phalloidin to F-actin. J. Biol. Chem. *269*, 14869–14871.

Campero, M., and Bostock, H. (2010). Unmyelinated afferents in human skin and their responsiveness to low temperature. Neurosci. Lett. *470*, 188–192.

Campero, M., Serra, J., and Ochoa, J.L. (1996). C-polymodal nociceptors activated by noxious low temperature in human skin. J. Physiol. *497*, 565–572.

Campero, M., Serra, J., Bostock, H., and Ochoa, J.L. (2001). Slowly conducting afferents activated by innocuous low temperature in human skin. J. Physiol. *535*, 855–865.

Carandini, M., and Churchland, A.K. (2013). Probing perceptual decisions in rodents. Nat. Neurosci. *16*, 824–831.

Caterina, M.J., Schumacher, M.A., Tominaga, M., Rosen, T.A., Levine, J.D., and Julius, D. (1997). The capsaicin receptor: a heat-activated ion channel in the pain pathway. Nature *389*, 816–824.

Caterina, M.J., Leffler, A., Malmberg, A.B., Martin, W.J., Trafton, J., Petersen-Zeitz, K.R., Koltzenburg, M., Basbaum, A.I., and Julius, D. (2000). Impaired nociception and pain sensation in mice lacking the capsaicin receptor. Science *288*, 306–313.

Chisholm, K.I., Khovanov, N., Lopes, D.M., La Russa, F., and McMahon, S.B. (2018). Large scale in vivo recording of sensory neuron activity with GCaMP6. eNeuro *5*, ENEURO.0417-17.2018.

Darian-Smith, I., Johnson, K.O., and Dykes, R. (1973). "Cold" fiber population innervating palmar and digital skin of the monkey: responses to cooling pulses. J. Neurophysiol. *36*, 325–346.

Darian-Smith, I., Johnson, K.O., LaMotte, C., Shigenaga, Y., Kenins, P., and Champness, P. (1979a). Warm fibers innervating palmar and digital skin of the monkey: responses to thermal stimuli. J. Neurophysiol. *42*, 1297–1315.

Darian-Smith, I., Johnson, K.O., LaMotte, C., Kenins, P., Shigenaga, Y., and Ming, V.C. (1979b). Coding of incremental changes in skin temperature by single warm fibers in the monkey. J. Neurophysiol. *42*, 1316–1331.

Dhaka, A., Murray, A.N., Mathur, J., Earley, T.J., Petrus, M.J., and Patapoutian, A. (2007). TRPM8 is required for cold sensation in mice. Neuron *54*, 371–378.

Dhaka, A., Earley, T.J., Watson, J., and Patapoutian, A. (2008). Visualizing cold spots: TRPM8-expressing sensory neurons and their projections. J. Neurosci. *28*, 566–575.

Dubner, R., Sumino, R., and Wood, W.I. (1975). A peripheral "cold" fiber population responsive to innocuous and noxious thermal stimuli applied to monkey's face. J. Neurophysiol. *38*, 1373–1389.

Filingeri, D. (2016). Neurophysiology of skin thermal sensations. Compr. Physiol. *6*, 1429.

Fleischer, E., Handwerker, H.O., and Joukhadar, S. (1983). Unmyelinated nociceptive units in two skin areas of the rat. Brain Res. *267*, 81–92.

Frenzel, H., Bohlender, J., Pinsker, K., Wohlleben, B., Tank, J., Lechner, S.G.S.G., Schiska, D., Jaijo, T., Rüschendorf, F., Saar, K., et al. (2012). A genetic basis for mechanosensory traits in humans. PLoS Biol. *10*, e1001318.

González, A., Ugarte, G., Restrepo, C., Herrera, G., Piña, R., Gómez-Sánchez, J.A., Pertusa, M., Orio, P., and Madrid, R. (2017). Role of the excitability brake potassium current IKD in cold allodynia induced by chronic peripheral nerve injury. J. Neurosci. *37*, 3109–3126.

Griffith, T.N., Docter, T.A., and Lumpkin, E.A. (2019). Tetrodotoxin-sensitive sodium channels mediate action potential firing and excitability in menthol-sensitive Vglut3-lineage sensory neurons. J. Neurosci. *39*, 7086–7101.

Hallin, R.G., Torebjörk, H.E., and Wiesenfeld, Z. (1982). Nociceptors and warm receptors innervated by C fibres in human skin. J. Neurol. Neurosurg. Psychiatry *45*, 313–319.

Huang, S.M., Li, X., Yu, Y., Wang, J., and Caterina, M.J. (2011). TRPV3 and TRPV4 ion channels are not major contributors to mouse heat sensation. Mol. Pain *7*, 37.

Iggo, A. (1969). Cutaneous thermoreceptors in primates and sub-primates. J. Physiol. *200*, 403–430.

Knowlton, W.M., Palkar, R., Lippoldt, E.K., McCoy, D.D., Baluch, F., Chen, J., and McKemy, D.D. (2013). A sensory-labeled line for cold: TRPM8-expressing sensory neurons define the cellular basis for cold, cold pain, and cooling-mediated analgesia. J. Neurosci. *33*, 2837–2848.

Koltzenburg, M., Stucky, C.L., and Lewin, G.R. (1997). Receptive properties of mouse sensory neurons innervating hairy skin. J. Neurophysiol. *78*, 1841–1850.

LaMotte, R.H., and Campbell, J.N. (1978). Comparison of responses of warm and nociceptive C-fiber afferents in monkey with human judgments of thermal pain. J. Neurophysiol. *41*, 509–528.

Lee, H., Iida, T., Mizuno, A., Suzuki, M., and Caterina, M.J. (2005). Altered thermal selection behavior in mice lacking transient receptor potential vanilloid 4. J. Neurosci. *25*, 1304–1310.

Lewin, G.R., and Mendell, L.M. (1994). Regulation of cutaneous C-fiber heat nociceptors by nerve growth factor in the developing rat. J. Neurophysiol. *71*, 941–949.

Lynn, B., and Carpenter, S.E. (1982). Primary afferent units from the hairy skin of the rat hind limb. Brain Res. *238*, 29–43.

Macmillan, N.A., and Kaplan, H.L. (1985). Detection theory analysis of group data: estimating sensitivity from average hit and false-alarm rates. Psychol. Bull. *98*, 185–199.

McKemy, D.D. (2013). The molecular and cellular basis of cold sensation. ACS Chem. Neurosci. *4*, 238–247.

McKemy, D.D., Neuhausser, W.M., and Julius, D. (2002). Identification of a cold receptor reveals a general role for TRP channels in thermosensation. Nature *416*, 52–58.

Milenkovic, N., Wetzel, C., Moshourab, R., and Lewin, G.R. (2008). Speed and temperature dependences of mechanotransduction in afferent fibers recorded from the mouse saphenous nerve. J. Neurophysiol. *100*, 2771–2783.

Milenkovic, N., Zhao, W.-J.W.-J., Walcher, J., Albert, T., Siemens, J., Lewin, G.R., and Poulet, J.F.A. (2014). A somatosensory circuit for cooling perception in mice. Nat. Neurosci. *17*, 1560–1566.

Moqrich, A., Hwang, S.W., Earley, T.J., Petrus, M.J., Murray, A.N., Spencer, K.S., Andahazy, M., Story, G.M., and Patapoutian, A. (2005). Impaired thermosensation in mice lacking TRPV3, a heat and camphor sensor in the skin. Science *307*, 1468–1472.

Pogorzala, L.A., Mishra, S.K., and Hoon, M.A. (2013). The cellular code for mammalian thermosensation. J. Neurosci. *33*, 5533–5541.

Ran, C., Hoon, M.A., and Chen, X. (2016). The coding of cutaneous temperature in the spinal cord. Nat. Neurosci. *19*, 1201–1209.

Schepers, R.J., and Ringkamp, M. (2010). Thermoreceptors and thermosensitive afferents. Neurosci. Biobehav. Rev. *34*, 177–184.

Shea, V.K., and Perl, E.R. (1985). Sensory receptors with unmyelinated (C) fibers innervating the skin of the rabbit's ear. J. Neurophysiol. *54*, 491–501.

Song, K., Wang, H., Kamm, G.B., Pohle, J., Reis, F. de C., Heppenstall, P., Wende, H., and Siemens, J. (2018). The TRPM2 channel is a hypothalamic heat sensor that limits fever and can drive hypothermia. Science *353*, 1393–1398.

Stevens, J.C., and Choo, K.K. (1998). Temperature sensitivity of the body surface over the life span. Somatosens. Mot. Res. *15*, 13–28.

Stevens, J.C., Marks, L.E., and Simonson, D.C. (1974). Regional sensitivity and spatial summation in the warmth sense. Physiol. Behav. *13*, 825–836.

Susser, E., Sprecher, E., and Yarnitsky, D. (1999). Paradoxical heat sensation in healthy subjects: peripherally conducted by A delta or C fibres? Brain A. J. Neurol. *122*, 239–246.

Takashima, Y., Daniels, R.L., Knowlton, W., Teng, J., Liman, E.R., and McKemy, D.D. (2007). Diversity in the neural circuitry of cold sensing revealed by genetic axonal labeling of transient receptor potential melastatin 8 neurons. J. Neurosci. *27*, 14147–14157.

Tan, C.-H., and McNaughton, P.A. (2016). The TRPM2 ion channel is required for sensitivity to warmth. Nature *536*, 460–463.

Togashi, K., Hara, Y., Tominaga, T., Higashi, T., Konishi, Y., Mori, Y., and Tominaga, M. (2006). TRPM2 activation by cyclic ADP-ribose at body temperature is involved in insulin secretion. EMBO J. *25*, 1804–1815.

Van Hees, J., and Gybels, J. (1981). C nociceptor activity in human nerve during painful and non painful skin stimulation. J. Neurol. Neurosurg. Psychiatry *44*, 600–607.

Vandewauw, I., De Clercq, K., Mulier, M., Held, K., Pinto, S., Van Ranst, N., Segal, A., Voet, T., Vennekens, R., Zimmermann, K., et al. (2018). A TRP channel trio mediates acute noxious heat sensing. Nature *555*, 662–666.

Walcher, J., Ojeda-Alonso, J., Haseleu, J., Oosthuizen, M.K., Rowe, A.H., Bennett, N.C., and Lewin, G.R. (2018). Specialized mechanoreceptor systems in rodent glabrous skin. J. Physiol. *596*, 4995–5016.

Wang, F., Bélanger, E., Côté, S.L., Desrosiers, P., Prescott, S.A., Côté, D.C., and De Koninck, Y. (2018). Sensory afferents use different coding strategies for heat and cold. Cell Rep. *23*, 2001–2013.

Wetzel, C., Pifferi, S., Picci, C., Gök, C., Hoffmann, D., Bali, K.K., Lampe, A., Lapatsina, L., Fleischer, R., Smith, E.S.J., et al. (2017). Small-molecule inhibition of STOML3 oligomerization reverses pathological mechanical hypersensitivity. Nat. Neurosci. *20*, 209–218.

Yamamoto, S., Shimizu, S., Kiyonaka, S., Takahashi, N., Wajima, T., Hara, Y., Negoro, T., Hiroi, T., Kiuchi, Y., Okada, T., et al. (2008). TRPM2-mediated Ca2+ influx induces chemokine production in monocytes that aggravates inflammatory neutrophil infiltration. Nat. Med. 738–747.

Yarmolinsky, D.A., Peng, Y., Pogorzala, L.A., Rutlin, M., Hoon, M.A., and Zuker, C.S. (2016). Coding and plasticity in the mammalian thermosensory system. Neuron *92*, 1079–1092.

Yarnitsky, D., and Ochoa, J.L. (1991). Warm and cold specific somatosensory systems. Psychophysical thresholds, reaction times and peripheral conduction velocities. Brain A. J. Neurol. *114*, 1819–1826.

Yudin, Y., Lutz, B., Tao, Y.X., and Rohacs, T. (2016). Phospholipase C δ4 regulates cold sensitivity in mice. J. Physiol. *594*, 3609–3628.

Zimmermann, K., Hein, A., Hager, U., Kaczmarek, J.S., Turnquist, B.P., Clapham, D.E., and Reeh, P.W. (2009). Phenotyping sensory nerve endings in vitro in the mouse. Nat. Protoc. *4*, 174–196.

Zimmermann, K., Lennerz, J.K., Hein, A., Link, A.S., Kaczmarek, J.S., Delling, M., Uysal, S., Pfeifer, J.D., Riccio, A., and Clapham, D.E. (2011). Transient receptor potential cation channel, subfamily C, member 5 (TRPC5) is a cold-transducer in the peripheral nervous system. Proc. Natl. Acad. Sci. USA *108*, 18114–18119.

STAR★METHODS

KEY RESOURCES TABLE

REAGENT or RESOURCE	SOURCE	IDENTIFIER
Chemicals, Peptides, and Recombinant Proteins		
PBMC TRPM8 blocker	Focus Biomolecules	Cat#10-1413
Toluidine Blue	Roth	Cat#0300.2
Uranyl Acetate	Serva	Cat#77870
Lead Citrate	Leica, Ultrastain 2	Cat#16705530
Experimental Models: Organisms/Strains		
Mouse: *Trpv1*$^{-/-}$ B6.129X1-Trpv1^{tm1Jul}	The Jackson Laboratory	JAX 003770
Mouse: *Trpm2*$^{-/-}$	Yamamoto et al., 2008	N/A
Mouse: *Trpm8*$^{-/-}$ B6.129P2-Trpm8^{tm1Jul}	The Jackson Laboratory	JAX 005693
Mouse: Trpv1$^{-/-}$*Trpm3*$^{-/-}$*Trpa1*$^{-/-}$ triple knockout (TKO) mice	Vandewauw et al., 2018	N/A
Mouse: C57BL/6J	The Jackson Laboratory	JAX 000664
Software and Algorithms		
Python	Python Software Foundation	https://www.python.org/
LabVIEW	National Instruments	https://www.ni.com/en-us.html
Prism 5.0 / 6.0	GraphPad Software	https://www.graphpad.com/scientific-software/prism/
OpenOffice Calc	Apache Software Foundation	https://www.openoffice.org/product/calc.html
Spike2	Cambridge Electronic Design Limited	http://ced.co.uk/products/spkovin

LEAD CONTACT AND MATERIALS AVAILABILITY

As Lead Contact, Gary Lewin will fulfill any requests for further information, resources or reagents. Please contact glewin@mdc-berlin.de. No new reagents or mouse lines were generated in this study.

EXPERIMENTAL MODEL AND SUBJECT DETAILS

Animals

All experiments were approved by the Berlin animal ethics committee and carried out in accordance with European animal welfare law. Adult Wild-type C57Bl6/J mice and transgenic mice were used. Both male and female mice were used in this study, but no obvious differences were observed between sexes. All mice were given *ad libitum* access to food and water, except in for prior to behavioral testing (see below). The following strains of transgenic mice were used: 1) *trpv1*$^{-/-}$ mice on a mixed background, from Jackson Laboratories (B6.129X1-Trpv1^{tm1Jul}) (Caterina et al., 2000). 2) *Trpm2*$^{-/-}$ mice on a mixed background (129/SvJ and C57Bl6/N), backcrossed with C57Bl6/J mice for several generations, kindly donated by Yasuo Mori, Kyoto University (Yamamoto et al., 2008). 3) *Trpm8*$^{-/-}$ mice on a mixed background, from Jackson Laboratories (B6.129P2-Trpm8^{tm1Jul}) (Bautista et al., 2007). 4). The *trpv1:trpa1:trpm3*$^{-/-}$ triple knockout mice on a C57BL/6J background were generated by Thomas Voets and Joris Vriens and made available for this study (Vandewauw et al., 2018). All mice were maintained on a 12h light/ 12h dark cycle.

METHOD DETAILS

Head implanting of mice for behavioral training

Mice were anesthetized with isoflurane (3%–4% initiation and 1.5%–2% maintenance in O_2) and injected subcutaneously with Metamizol (200 mg per kg of body weight). Temperature of mice was monitored with a rectal probe and kept at 37°C using a heating pad. A light metal support was implanted onto the skull with glue (UHU dent) and dental cement (Paladur). Mice were then placed in their home cage with Metamizol (200 mg/ml) in the drinking supply 1-3 days.

CellPress

Behavioral training

Initially, head implanted mice were habituated to head-restraint in the behavioral setup for three days with increasing restriction times (15, 30 and 60 mins). During the second and third habituation sessions, the right forepaw was fixed to the ground with medical tape, in order to habituate the mice to paw-restraint.

Next, mice were water restricted and they underwent two "pairing" sessions in consecutive days. In these, water rewards were given from a water spout paired to presentation of the thermal stimulus in the forepaw (via an 3x3 or 8x8 mm Peltier element stimulator); to build an association between stimulus and reward. Each session lasted 1 hour approximately.

Mice that had undergone habituation and pairing started behavioral training. During training, mice only got a water reward (4-7 μl) from the spout when they licked it during a timeout upon start of the stimulus (3.5 s). Catch trials (where no stimulus is presented but licks are counted as false alarms) were included, interleaved, as 50% of the total trials.

Performance was assessed by counting hits and false alarms. All trials were delivered at randomized time intervals between 3 and 30 s. A training session consisted of about 100 trials (50 stimulus + 50 catch). Baseline temperature was 32°C, and stimuli consisted on an initial ramp to reach goal temperature (0.5 s), a hold phase (3 s) and a phase in which temperature returned to baseline (0.5 s). it was increased or decreased in 10°C during stimuli. In threshold experiments, stimulus amplitude was reduced every day (e.g., 6, 4, 2, 1, 0.5°C).

For sound training of *Trpm8*$^{-/-}$ mice, a magnetic buzzer generated a sound stimulus of roughly 40 dB SPL that lasted for as long as the thermal stimulus. In the mechanical stimulation training, a Piezo stimulator produced a 3.5 s long single contact with the glabrous skin of the forepaw, and mice were rewarded when they licked within a time window of the same length as the thermal training.

Skin-nerve preparation and sensory afferent recordings

Cutaneous sensory fiber recordings were performed using the *ex vivo* skin nerve preparation. Mice were euthanized by CO_2 inhalation for 2-4 min followed by cervical dislocation. In experiments using *Trp* knockout mice and C57/Bl6J control mice, the saphenous nerve and shaved hairy skin of the hind limb were dissected free. In forepaw experiments, the forepaw glabrous skin and innervating medial and ulnar nerves were dissected in a separate group of C57/Bl6J control mice. Skin and nerve samples were placed in an organ bath of 32°C perfused with a synthetic interstitial fluid (SIF buffer): 123mM NaCl, 3.5mM KCl, 0.7mM $MgSO_4$, 1.7mM NaH_2PO_4, 2.0mM $CaCl_2$, 9.5 mM sodium gluconate, 5.5mM glucose, 7.5mM sucrose and 10mM HEPES (pH7.4). The saphenous/medial and ulnar nerves were placed in an adjacent chamber in mineral oil, where fine filaments were teased from the nerve and placed on the recording electrode.

The receptive fields of individual thermosensory units were identified by pipetting hot (~48°C) and cold (~5°C) SIF buffer onto the surface of the skin. Electrical stimuli (1Hz, square pulses of 50-500ms) were delivered to unit receptive fields to classify them as C-fibers (velocity < 1.2 m/s), A-delta fibers (1.2-10 m/s) or A-beta fibers (> 10 m/s). To test mechanosensitivity of units, four 3 s duration ramp and hold mechanical stimuli of increasing amplitude (20-400mN) were delivered using a computer controlled nanomotor® (Kleindieck, Germany).

To test thermal responses of units, a computer controlled Peltier device with a 3x3mm contact point (custom device built by Yale School of Medicine Instrumentation Repair and Design) was placed on the center of the unit receptive field and a series of thermal stimuli were applied. In hairy hindpaw skin experiments, a heat ramp from 32 to 48°C (1°C/s) and a cold ramp from 32 to 12°C (1°C/s) was used. Average responses were obtained from three heat and cold ramps, with 2 minute intervals between each stimuli. In forepaw experiments, thermosensory unit receptive fields were stimulated with warm ramps which matched behavioral experiments: 0.5 s ramp, 3 s hold, and 0.5 s ramp to baseline. 32-42°C warm ramps and 32-22°C cold ramps were given, and if units responded to these stimuli then a series of warm and/or cool ramps were given which decreased the amplitude by 2°C (e.g., 32-40°C, 32-38°C etc), followed by 32-33°C and 32-32.5 heat ramps, and/or 32-31°C and 32-31.5°C cool ramps. Thermal ramps were repeated 3-7 times, depending on the recording, to create average cell responses. Sensory fiber receptive fields were also stimulated using 1°C/s 32-48°C heat and 32-12°C cold ramps. Cells which exhibited signs of wind up or spontaneous activity after multiple stimulations were discarded from analysis.

Transdermal injections in the forepaw

Mice that had been head implanted and trained (6 sessions) to report non-painful thermal stimuli in the forepaw were briefly anesthetized with isoflurane (3%–4% initiation and 1.5%–2% maintenance in O_2). Once the pain reflexes were absent due to the anesthesia, 10 μL of solution were injected transdermally into the plantar side of the right forepaw, using a syringe of gauge 30G (0.3mm). Afterward, mice recovered from anesthesia. 15 minutes after the injection, all mice were active and were tested in the thermal perception task. As in all behavioral experiments described here, thermal stimuli were delivered to the right forepaw.

To control for the possible effects of the injection procedure and the anesthesia, mice were injected in two occasions in different days: once with a solution in which the TRPM8 antagonist PBMC was absent (DMSO control); and once with a solution containing the drug (PBMC group). The injected solutions consisted of 4 μL of DMSO with 0.1 mg of PBMC diluted in 6 μL of saline (PBMC injection) and 4 μL of DMSO in 6 μL of saline (DMSO control).

QUANTIFICATION AND STATISTICAL ANALYSIS

Analysis of behavior

Licks were recorded with a sensor at the tip of the water reward spout. A thermocouple wire placed at the interface Peltier-forepaw skin measured the temperature during the training sessions. In stimulus trials, a hit was counted when there was a lick within the window of opportunity (3.5 s) after the start of the stimulus. During catch trials, a false alarm took place when there was a lick during an equally long window of opportunity.

To assess whether mice successfully learnt the detection task, hit rates were compared to false alarm rates within the same training session. Latencies to respond to stimuli were quantified and compared between groups as an additional measure.

To quantify performance in the detection tasks, we used d’ (sensitivity index) instead of the percentage of correct trials, in order to take into account bias in the licking criterion (Carandini and Churchland, 2013). To calculate d’, the following formula was used: d’ = z(h) – z(fa), where z(h) and z(fa) are the normal inverse of the cumulative distribution function of the hit and false alarm rates, respectively. To avoid infinity d’ values, when all trials were reported (rate = 1) or none of them was (rate = 0), the rates were replaced by (1-1/2N) or (1/2N), respectively, where N is the number of trials the stimulus was presented (Macmillan and Kaplan, 1985).

The z scores for hit and false alarm rates were calculated with OpenOffice Calc (Apache Software Foundation) using the function NORMINV.

Behavioral data was collected used custom-written routines in Lab View at 1 kHz sampling rate, and custom-written Python scripts were used for analysis.

Analysis of skin-nerve recordings

Cutaneous forepaw and hindpaw thermosensory units were categorized based on their conduction velocity and responses to thermal and mechanical stimuli.

Single unit recording thermal data points represent a mean response of > 3 stimuli. Thermal and mechanical thresholds of units were calculated as the temperature or mechanical amplitude required to elicit the first action potential. In forepaw experiments, heat and cold-evoked firing activity was compared between different fiber populations e.g., C-mechanoheat (C-MH) versus C-mechanoheatcold (C-MHC). In hindpaw experiments, population responses of units recorded from wild-type control and *trp* knockout mice were statistically compared. Spike histogram graphs represent pooled data from multiple responses within and between C-fiber recordings in different animals.

Statistical tests

Statistical analyses were performed with GraphPad Prism 5.0/6.0 and Python. Statistical tests for significance are stated in the text, and include two-way repeated-measures ANOVA with Bonferroni’s post hoc test, Student t test, Mann Whitney test and Wilcoxon matched pairs test. Kolmogorov-Smirnov test was used to assess normality of the data. Asterisks in figures indicate statistical significance: *$p < 0.05$, **$p < 0.01$, ***$p < 0.001$.

DATA AND CODE AVAILABILITY

The datasets/code generated in the current study have not been uploaded to a public repository because of large file size, but are available upon reasonable request.

Neuron, Volume *106*

Supplemental Information

The Sensory Coding of Warm Perception

Ricardo Paricio-Montesinos, Frederick Schwaller, Annapoorani Udhayachandran, Florian Rau, Jan Walcher, Roberta Evangelista, Joris Vriens, Thomas Voets, James F.A. Poulet, and Gary R. Lewin

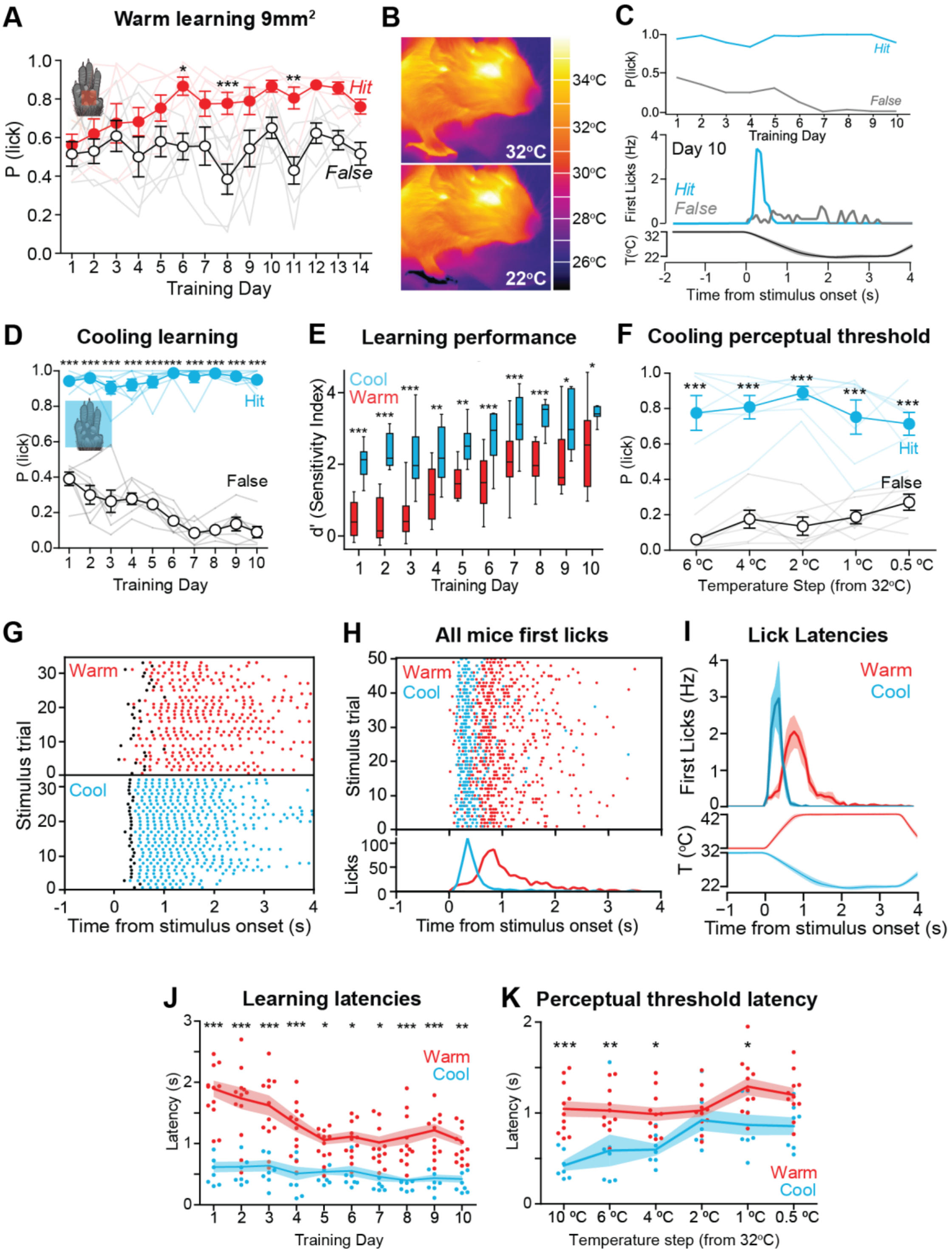

Figure S1 – Related to Figure 1 Comparison Warm vs Cool

(A) Wild type mice did not learn to reliably report warm stimuli of 32-42°C (delivered by a 3x3mm Peltier) after 14 training sessions (n = 7; two-way repeated measures ANOVA with Bonferroni post-hoc tests). (B) Wild type mice were trained to report cool stimuli of 32-22 °C delivered to the right forepaw. (C) Representative learning curve (top) and lick latency distribution at training day 10 (bottom) of a cool-trained mouse using an 8x8 mm Peltier element. (D) Wild type mice learnt to report cool stimuli of 32-22°C(delivered by a 8x8mm Peltier) from the 1st training session (n = 7; two-way repeated measures ANOVA with Bonferroni post-hoc tests). (E) Sensitivity (d’) was higher for cool- than for warm-trained mice across training sessions (n = 7 cool, n = 12 warm; two-way repeated measures ANOVA with Bonferroni post-hoc tests). (F) Decreasing stimulus amplitude over

consecutive training sessions revealed a perceptual threshold of 0.5°C (n = 6; two-way repeated measures ANOVA with Bonferroni post-hoc tests). (G) Lick latencies of a representative warm-trained (top) and cool-trained (bottom) mice on the last training session. The first lick to respond to each stimulus (latency) is shown in black, and the rest of the licks are shown in red (warm) or blue (cool). (H) Plot of all lick latencies from all mice on the last training session for cool and warm shows a higher spread and longer latency of the licks in warm-trained mice. (I) First lick latency PSTH of all warm- and cool-trained mice on their fastest session (chosen from sessions of good performance, with d' > 1.5) (top). Average temperature trace during warm and cool detection sessions show very similar Peltier dynamics during cooling and warming (bottom). (J) Session average lick latencies were slower for warm than for cool stimuli across training sessions (n = 12 warm, n = 7, two-way repeated measures ANOVA with Bonferroni post-hoc tests).

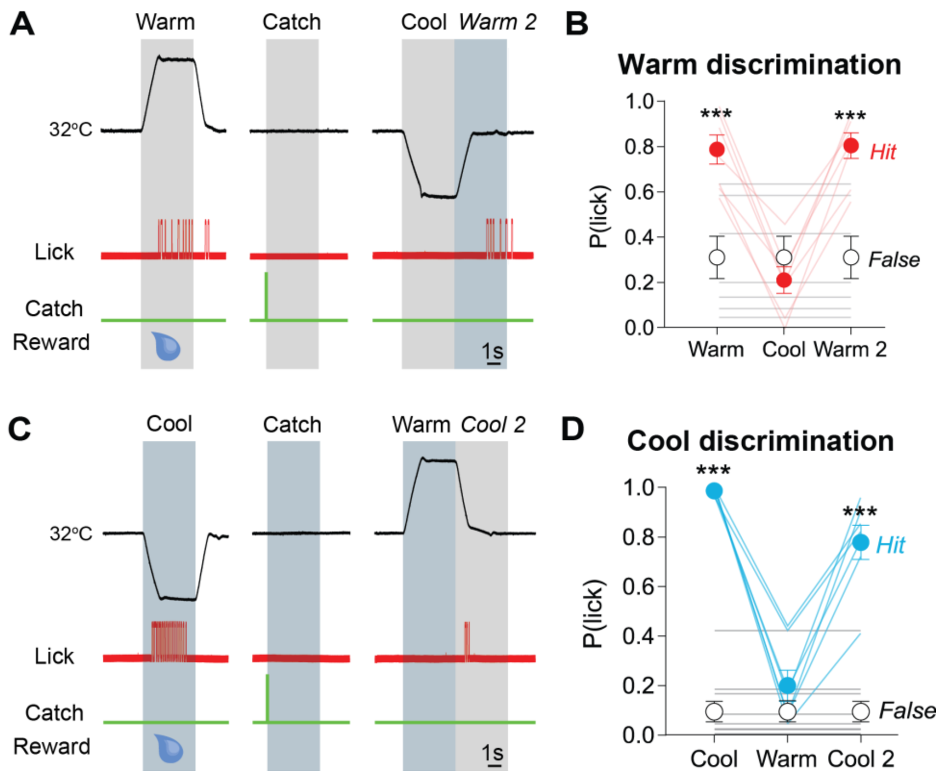

Figure S2 – Related to Figure 1 Discrimination task

(A) Scheme of the thermal discrimination task for warm-trained mice. Cool trials were introduced, and no reward was given if mice licked during cool stimuli. Licks were also assessed during the warming phase of the right after the cool stimulus ("Warm 2"). (B) Warm-trained mice licked the sensor during both warm types, but not during cool stimuli (n = 7, hit vs false; two-way repeated measures ANOVA with Bonferroni post-hoc tests).

(C) Scheme of the thermal discrimination task for cool-trained mice. Warm trials were introduced, and no reward was given if mice licked during warm stimuli. Licks were also assessed during the cool phase right after the cool stimulus ("Cool 2"). (D) Cooling-trained mice could correctly discriminate cooling from warm, and reported cool regardless of the absolute temperature (n = 7; hit vs false two-way repeated measures ANOVA with Bonferroni post-hoc tests). *$P < 0.05$, **$P < 0.01$, ***$P < 0.001$. Data = mean ± SEM.

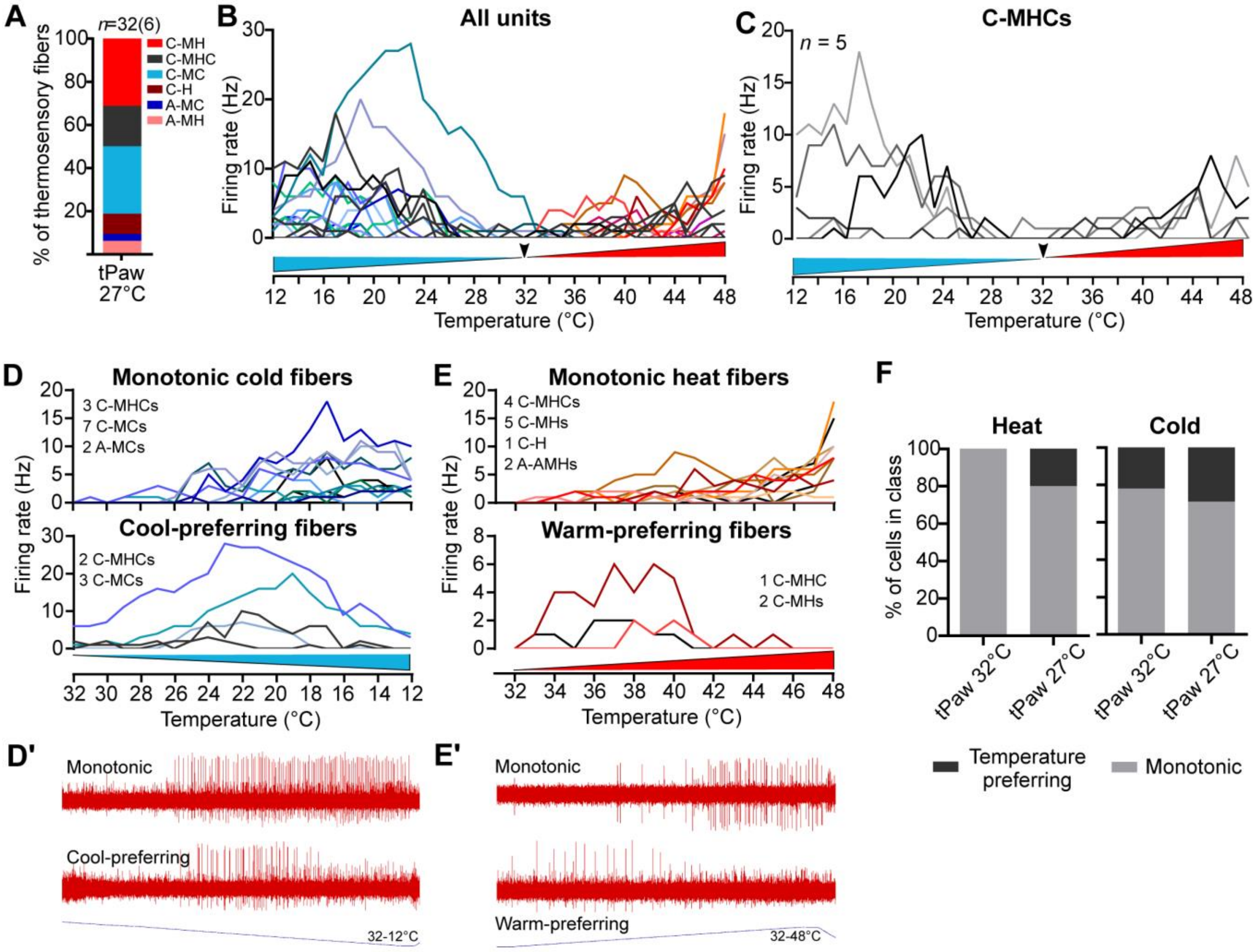

Figure S3 – Related to Figure 2 Additional skin-nerve data at bath 27˚C

(A) Proportions of thermosensory C-fibers in control mice recorded from the forepaw skin with bath temperature at 27 ˚C. Total animals recorded from shown in brackets. (B) Firing activity of all individual fibers during 32-48ºC and 32-12ºC ramps. Arrow marks stimulus starting point. (C) Firing activity of all individual C-mechanoheatcold (C-MHC) fibers during 32-48˚C and 32-12˚C ramps. (D) Cold-sensitive fibers were either monotonic (top) and increased firing activity linearly during 32-12˚C ramp, or were cool-preferring (bottom) and preferentially fired during the cool phase of the ramp. (D') Example traces recorded from a monotonic (top) and a cool-preferring (bottom) fiber. (E) Heat-sensitive fibers were either monotonic (top) and increased firing activity linearly during 32-48 ˚C ramp, or were warm-preferring (bottom) and preferentially fired during the warm phase of the ramp. (E') Example traces recorded from a monotonic (top) and a warm-preferring (bottom) fiber. (F) Proportions of heat and cold fiber subtypes recorded when the temperature of the paw was set to 32 ˚C or 27˚C.

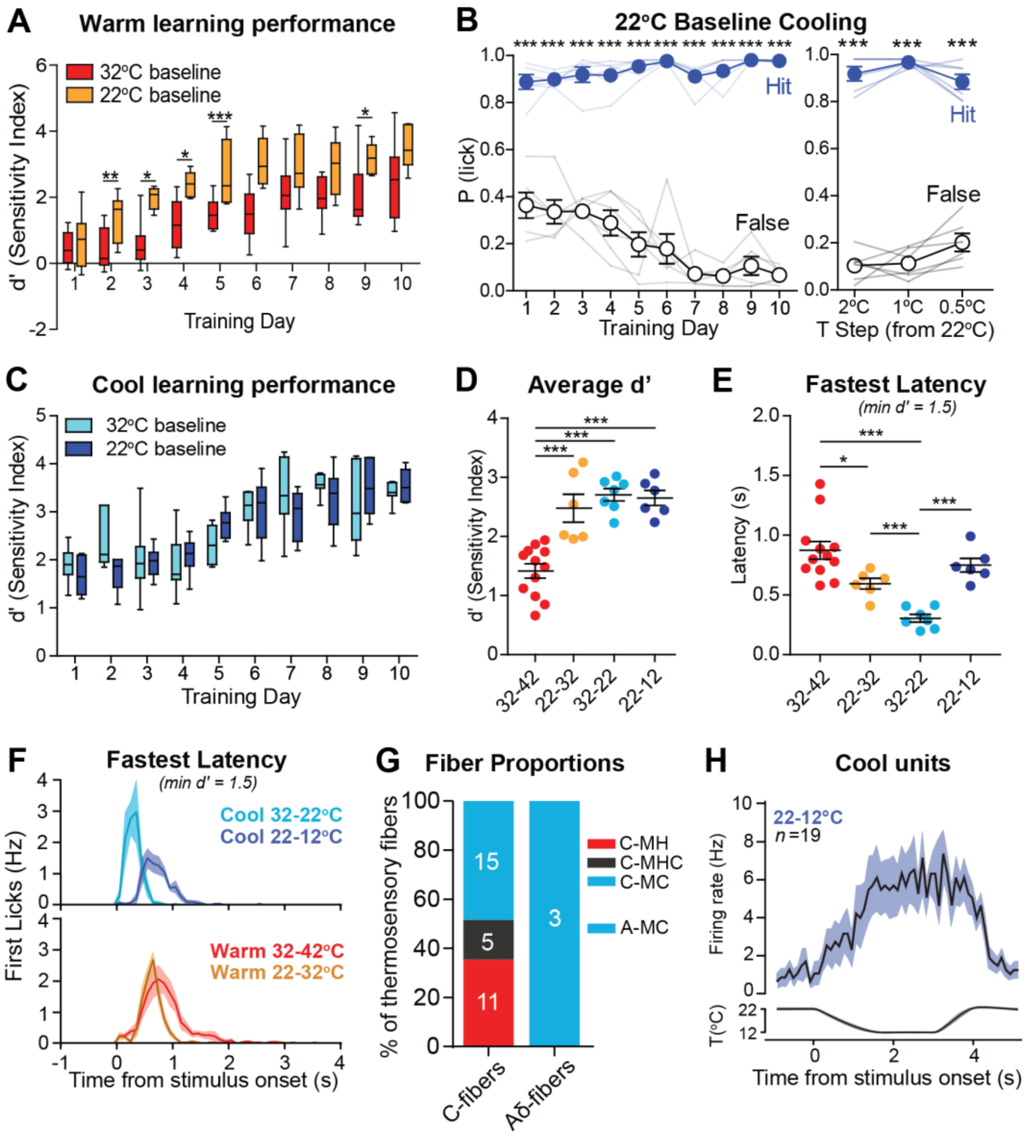

Figure S4 – Related to Figure 3 Additional data 22˚C baseline

(A) Sensitivity (d') of WT mice trained to report warm of 32-42˚C (n = 12) and 22-32˚C (n = 6). Mice trained to report warm of 22-32˚C had an overall slightly better performance than mice trained at 32-42˚C (two-way repeated measures ANOVA with Bonferroni post-hoc). (B) WT mice could report cool of 22-12ºC (n = 6), as well as cool stimuli of 0.5˚C starting from 22˚Cbaseline (n = 6) (hit vs false two-way repeated measures ANOVA with Bonferroni post-hoc). (C) Sensitivity (d') of WT mice trained to report cool of 32-22˚C (n = 7) was overall very similar to that of mice trained to report cool of 22-12˚C (n = 6) (two-way repeated measures ANOVA with Bonferroni post-hoc). (D) Average sensitivity (d') across all training sessions was lower for mice trained to report warming at 32-42˚C (n = 12, d' = 1.42 ± 0.12) than for warming at 22-32˚C (n = 6, d' = 2.48 ± 0.24, p = 0.0004 vs 32-42˚C), cool at 32-22˚C (n = 7, d' = 2.71 ± 0.11, p < 0.0001 vs 32-42˚C) and cooling at 22-12˚C (n = 6, d' = 2.65 ± 0.13, p < 0.0001 vs 32-42˚C). (E) The fastest latency achieved across all training sessions (with good performance, d' at least 1.5) was slower for WT mice trained to report warm at 32-42˚C (n = 12, mean 0.87 ± 0.07s) than for warm at 22-32˚C (n = 6, mean 0.59 ± 0.04s, p = 0.023 vs 32-42˚C). However, cool steps of 32-22˚C (n = 7, mean 0.31 ± 0.03s) could be reported quicker than cool steps of 22-12˚C (n = 6, mean 0.75 ± 0.06s, p < 0.0001 vs 32-22˚C). Also, cool step at 32-22˚C could be reported faster than warm at 32-42˚C (p < 0.0001). (F) Mean hit and false alarm lick PSTHs of WT mice trained to report cool steps of either 32-22 (n = 7) or 22-12˚C (n = 6) (top), and warm steps of 32-42 (n = 12) or 22-32˚C (n = 6) (bottom). (G) Fiber proportions found in skin-nerve sensory afferent recordings of forepaw in the 22˚C Peltier baseline experiments (and tissue kept in a bath at 27˚C) (n = 34 fibers). Fibers were screened using a slow ramp between 12 and 42˚C. (H) PSTH of

sensory afferent action potential responses to 4-second cool steps at 22-12°C. *$P < 0.05$, **$P < 0.01$, ***$P < 0.001$. Data = mean ± SEM.

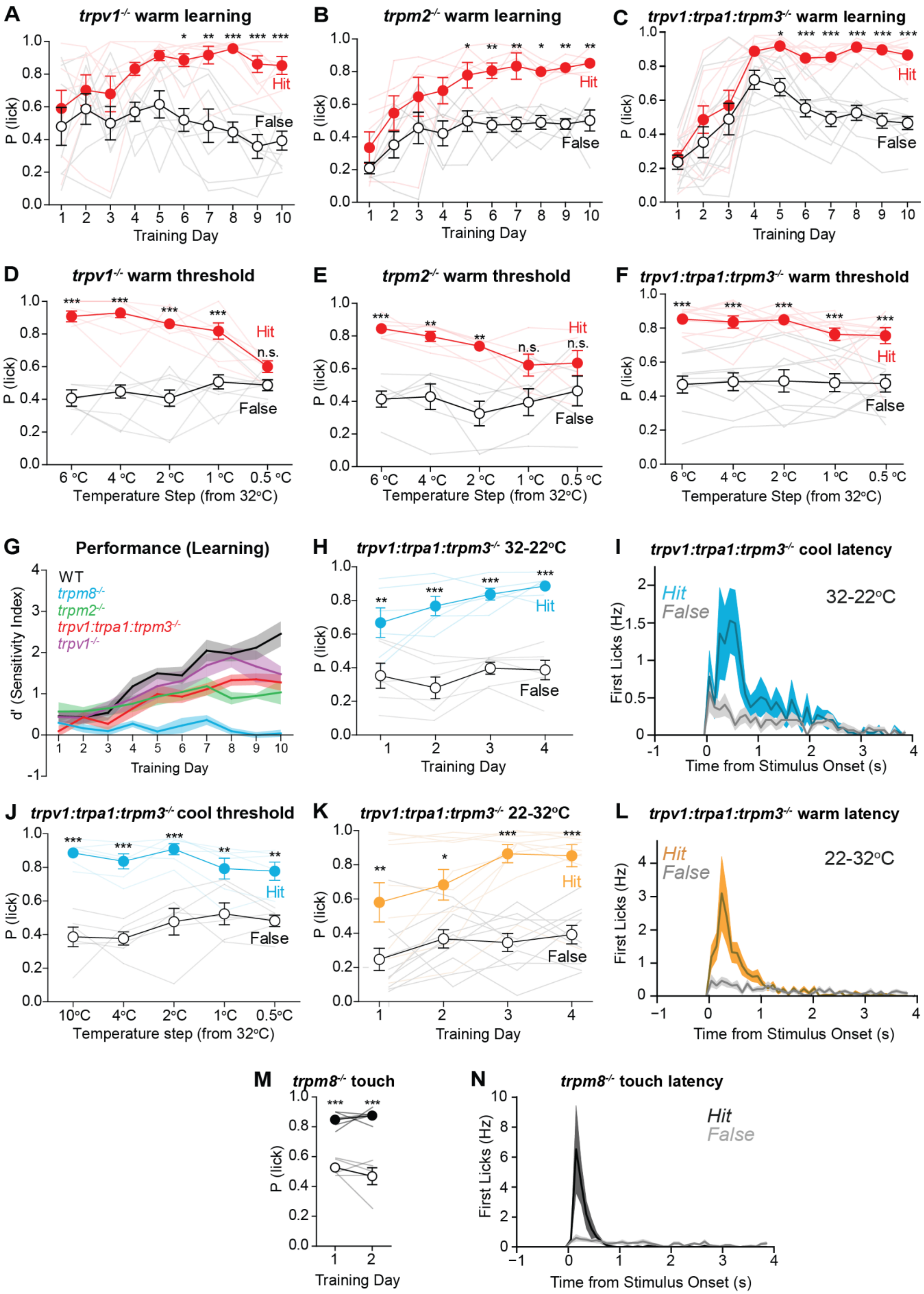
A
trpv1-/- warm learning
P (lick)
Training Day
Hit
False
* ** *** *** ***
B
trpm2-/- warm learning
* ** ** * ** **
C
trpv1:trpa1:trpm3-/- warm learning
* *** *** *** *** ***
D
trpv1-/- warm threshold
*** *** *** *** n.s.
Temperature Step (from 32°C)
6 °C 4 °C 2 °C 1 °C 0.5 °C
E
trpm2-/- warm threshold
*** ** ** n.s. n.s.
F
trpv1:trpa1:trpm3-/- warm threshold
*** *** *** *** ***
G
Performance (Learning)
d' (Sensitivity Index)
WT
trpm8-/-
trpm2-/-
trpv1:trpa1:trpm3-/-
trpv1-/-
H
trpv1:trpa1:trpm3-/- 32-22°C
** *** *** ***
I
trpv1:trpa1:trpm3-/- cool latency
32-22°C
First Licks (Hz)
Time from Stimulus Onset (s)
J
trpv1:trpa1:trpm3-/- cool threshold
*** *** *** ** **
10°C 4°C 2°C 1°C 0.5°C
Temperature step (from 32°C)
K
trpv1:trpa1:trpm3-/- 22-32°C
** * *** ***
L
trpv1:trpa1:trpm3-/- warm latency
22-32°C
M
trpm8-/- touch
*** ***
N
trpm8-/- touch latency

Figure S5 – Related to Figure 5 Additional *trp* data and WT Comparison

(A) *trpv1*$^{-/-}$ mice learnt to report warm stimuli of 32-42°C delivered to the forepaw (n = 8, hit vs false two-way repeated measures ANOVA with Bonferroni post-hoc tests). (B) *trpm2*$^{-/-}$ mice learnt to report warm stimuli of 32-42°C delivered to the forepaw (n = 6, hit vs false two-way repeated measures ANOVA with Bonferroni post-hoc tests). (C) *trpv1:trpa1:trpm3*$^{-/-}$ mice learnt to report warm stimuli of 32-42°C delivered to the forepaw (n = 10, hit vs false two-way repeated measures ANOVA with Bonferroni post-hoc tests). (D) *trpv1*$^{-/-}$ mice could report warm stimuli of as little as 1°C (n = 8, hit vs false two-way repeated measures ANOVA with Bonferroni post-hoc tests). (E) *trpm2*$^{-/-}$ mice could report warm stimuli of as little as 2°C (n = 6, hit vs false two-way repeated measures ANOVA with Bonferroni post-hoc tests). (F) *trpv1:trpa1:trpm3*$^{-/-}$ mice could also report very small warm stimuli as indicated by statistical differences between hit and false alarms, although false alarms were particularly high in this group (n = 10, hit vs false two-way repeated measures ANOVA with Bonferroni post-hoc tests). (G) Sensitivity (d') measurements of warm detection over training sessions for WT, *trpv1*$^{-/-}$, *trpm2*$^{-/-}$, *trpv1:trpa1:trpm3*$^{-/-}$ and *trpm8*$^{-/-}$ mice show that WT mice, as well as the heat-activated *trp* mutant lines had performances above chance level (d' = 0) but *trpm8*$^{-/-}$ mice remained close to d' = 0 across all sessions. (H) *trpv1:trpa1:trpm3*$^{-/-}$ mice learnt to report cool stimuli of 32-22°C delivered to the forepaw (n=6, hit vs false two-way repeated measures ANOVA with Bonferroni post-hoc tests). (I) First lick PSTH of *trpv1:trpa1:trpm3*$^{-/-}$ mice during session 4 of cool detection (32-22°C). (J) *trpv1:trpa1:trpm3*$^{-/-}$ mice could report tiny cool stimuli delivered to the forepaw (n = 6, hit vs false two-way repeated measures ANOVA with Bonferroni post-hoc tests). (K) *trpv1:trpa1:trpm3*$^{-/-}$ mice could report cool stimuli of 22-32°C delivered to the forepaw (n = 10, hit vs false two-way repeated measures ANOVA with Bonferroni post-hoc tests). (L) First lick PSTH of *trpv1:trpa1:trpm3*$^{-/-}$ mice during session 4 of 22-32°C warm detection. (M) Despite being unable to report warm stimuli, *trpm8*$^{-/-}$ mice could report tactile stimuli delivered to their forepaw (n = 5, hit vs false two-way repeated measures ANOVA with Bonferroni post-hoc tests). (N) First lick PSTH of *trpm8*$^{-/-}$ mice during session 2 of tactile detection shows that mice reported the tactile stimulus with high precision (n = 5). *P < 0.05, **P < 0.01, ***P < 0.001. Data = mean ± SEM.

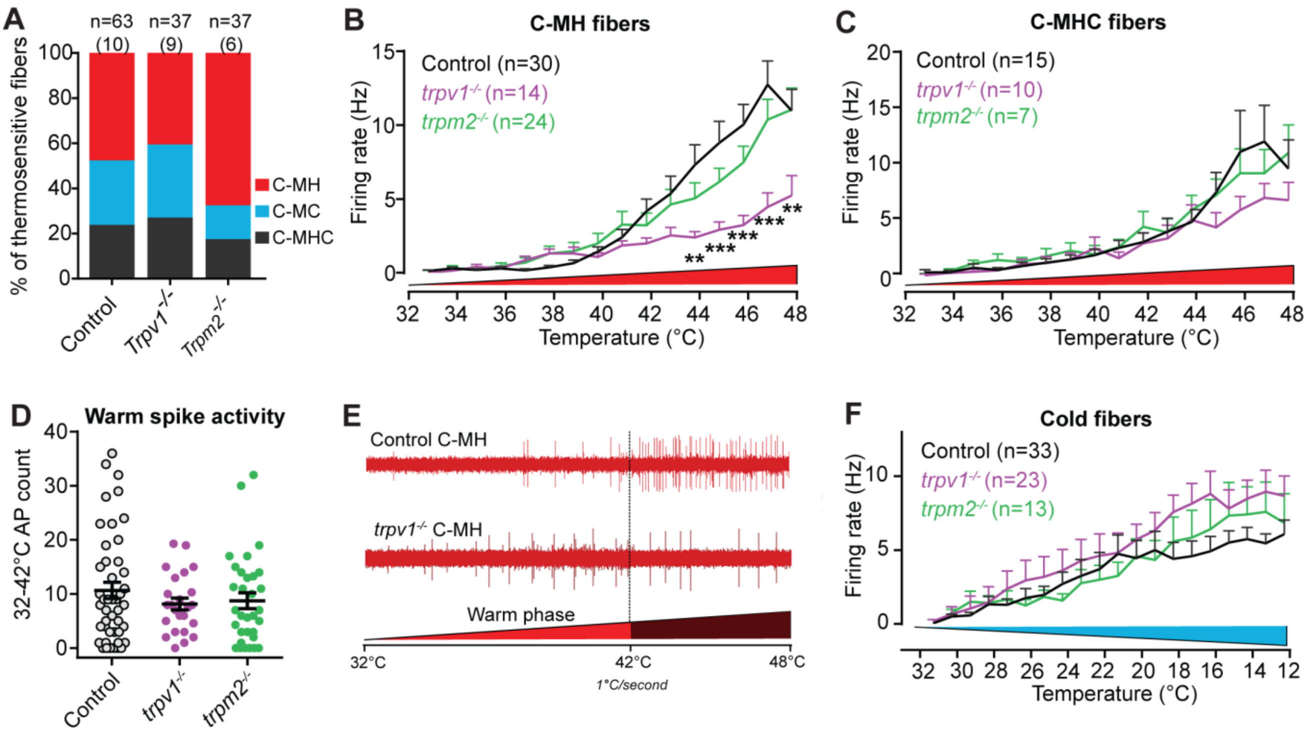

Figure S6 – Related to Figure 5 *trpv1*$^{-/-}$ and t*rpm2*$^{-/-}$ skin-nerve recordings

(A) Proportions of thermosensory C-fibers in control, *trpv1*$^{-/-}$ and t*rpm2*$^{-/-}$ mice recorded from the hindpaw skin with bath temperature at 32ºC. Total number of animals recorded from in each group shown in brackets. (B) Firing activity of C-mechanoheat (C-MH) fibers during 32-48˚C heat ramp. C-MH fibers recorded in *trpv1*$^{-/-}$ mice had significantly lower spike activity compared to control fibers from 43-48 ˚C (Repeated measures Two-way ANOVA with Bonferroni post-hoc test). (C) Firing activity of C-mechanoheatcold (C-MHC) fibers during 32-48 ˚C heat ramp. (D) Total spike activity of heat-evoked fibers during the warming phase of the ramp (32-42˚C). (E) Example recording traces from a control C-MH and a *trpv1*$^{-/-}$ C-MH fiber during heat ramp. (F) Firing activity of all cold-sensitive fibers during 32-12˚C cold ramp. **P < 0.01, ***P < 0.001. Data = mean ± SEM.

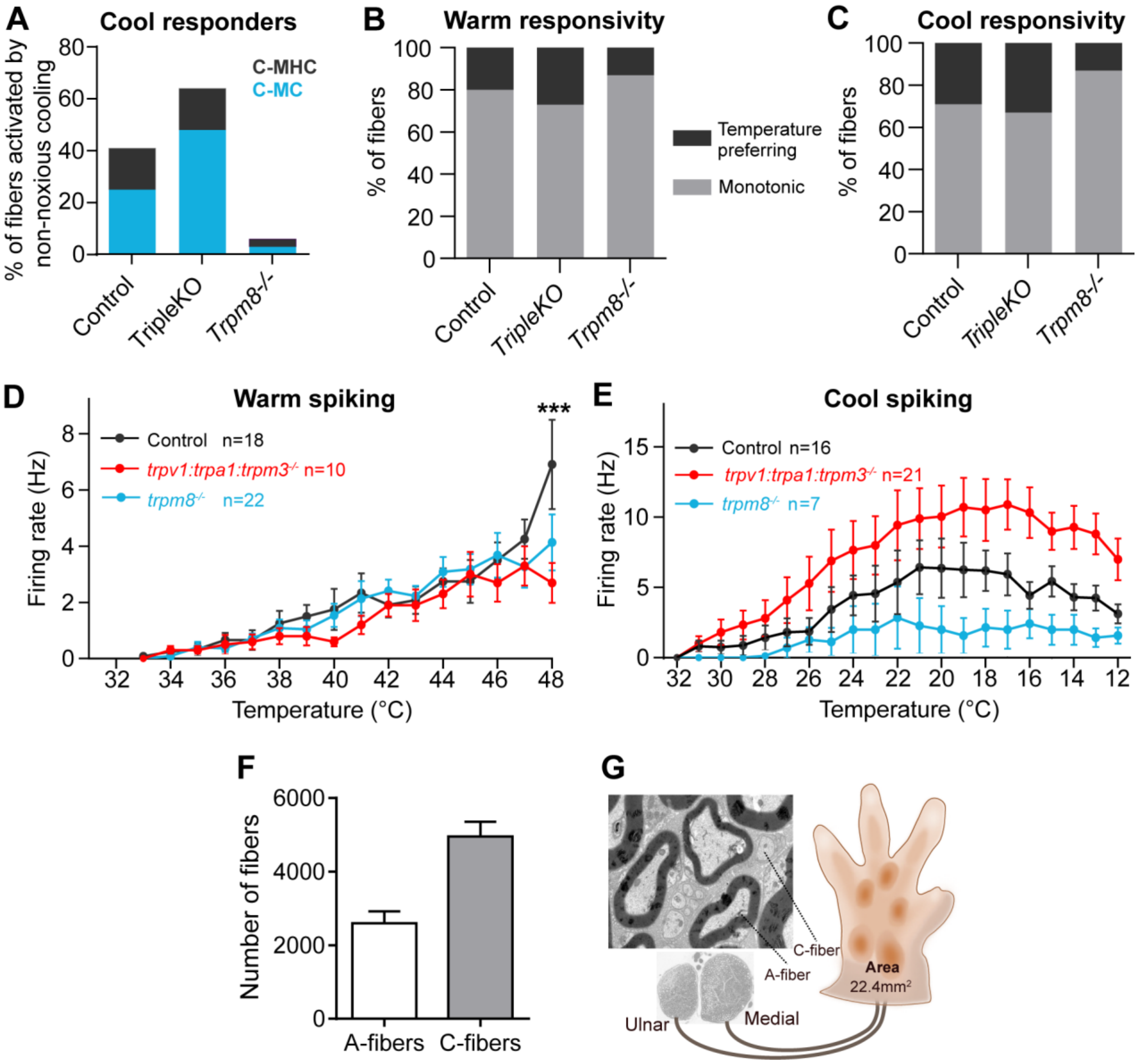

Figure S7 – Related to Figure 7 Additional t*rpm8*$^{-/-}$ and *trpv1:trpa1:trpm3*$^{-/-}$ recording data

(A) Proportions of fibers that were responsive to non-noxious cooling. (B) Proportions of heat-monotonic or warm-preferring units. (C) Proportions of cold-monotonic or cool-preferring units. (D) Firing activity of heat-responsive fibers during 32-48°C heat ramp. Fibers recorded in *trpv1:trpa1:trpm3*$^{-/-}$ mice had significantly lower spike activity compared to control fibers at 48°C (Repeated measures Two-way ANOVA with Bonferroni post-hoc test). (E) Firing activity of cold-responsive fibers during 32-12°C heat ramp. (F) Number of A-fibers and C-fibers that innervate the forepaw, estimated via electron microscopy (n = 4). (G) Example electron micrograph of afferent myelinated (A-type) and unmyelinated (C-type) fibers of the medial and ulnar nerves, which innervate the forepaw skin area. ***P < 0.001. Data = mean ± SEM.
